# Parameter optimization in S-system models

**DOI:** 10.1186/1752-0509-2-35

**Published:** 2008-04-16

**Authors:** Marco Vilela, I-Chun Chou, Susana Vinga, Ana Tereza R Vasconcelos, Eberhard O Voit, Jonas S Almeida

**Affiliations:** 1 Dept. Bioinformatics and Computational Biology, University of Texas M.D. Anderson Cancer Center, 1515 Holcombe Blvd, Houston, TX 77030, USA; 2 Dept. Biomedical Engineering, Georgia Institute of Technology and Emory University, 313 Ferst Drive, Atlanta, GA 30332, USA; 3 Instituto de Engenharia de Sistemas e Computadores: Investigação e Desenvolvimento (INESC-ID), R. Alves Redol 9, 1000-029 Lisboa, Portugal; 4 Dept. Computatinal and Applied Mathematics, Laboratório Nacional de Computação Científica, Petrópolis, Rio de Janeiro, Brazil; 5 Instituto de Tecnologia Química e Biológica, Universidade Nova de Lisboa, Rua da Quinta Grande 6, Apartado 127, 2780-156 Oeiras, Portugal

## Abstract

**Background:**

The inverse problem of identifying the topology of biological networks from their time series responses is a cornerstone challenge in systems biology. We tackle this challenge here through the parameterization of S-system models. It was previously shown that parameter identification can be performed as an optimization based on the decoupling of the differential S-system equations, which results in a set of algebraic equations.

**Results:**

A novel parameterization solution is proposed for the identification of S-system models from time series when no information about the network topology is known. The method is based on eigenvector optimization of a matrix formed from multiple regression equations of the linearized decoupled S-system. Furthermore, the algorithm is extended to the optimization of network topologies with constraints on metabolites and fluxes. These constraints rejoin the system in cases where it had been fragmented by decoupling. We demonstrate with synthetic time series why the algorithm can be expected to converge in most cases.

**Conclusion:**

A procedure was developed that facilitates automated reverse engineering tasks for biological networks using S-systems. The proposed method of eigenvector optimization constitutes an advancement over S-system parameter identification from time series using a recent method called *Alternating Regression*. The proposed method overcomes convergence issues encountered in alternate regression by identifying nonlinear constraints that restrict the search space to computationally feasible solutions. Because the parameter identification is still performed for each metabolite separately, the modularity and linear time characteristics of the alternating regression method are preserved. Simulation studies illustrate how the proposed algorithm identifies the correct network topology out of a collection of models which all fit the dynamical time series essentially equally well.

## Background

Metabolic and genetic time series have arisen as important sources of information about biological processes. However, the quantitative characterization of these processes from their temporal responses is not a trivial problem due to the complexity of typical biological networks and the multi-fold interdependencies among their components. Any effective method for this task needs to be able to filter out all possible quantitative information from observed time series and convert it into mathematical features that reliably characterize the true topology of the network, as well as its regulation. Biochemical System Theory (BST) [1-3] has been shown to provide a consistent mathematical framework for representing and analyzing biological systems. The S-system variant of BST represents the biological network as a set of differential equations in the general format

$${\dot{X}}_{i}=\alpha_{i}\prod_{j=1}^{M}X_{j}^{g_{ij}}-\beta_{i}\prod_{j=1}^{M}X_{j}^{h_{ij}},i=1,2,\cdots ,M.$$

**Figure 1:**
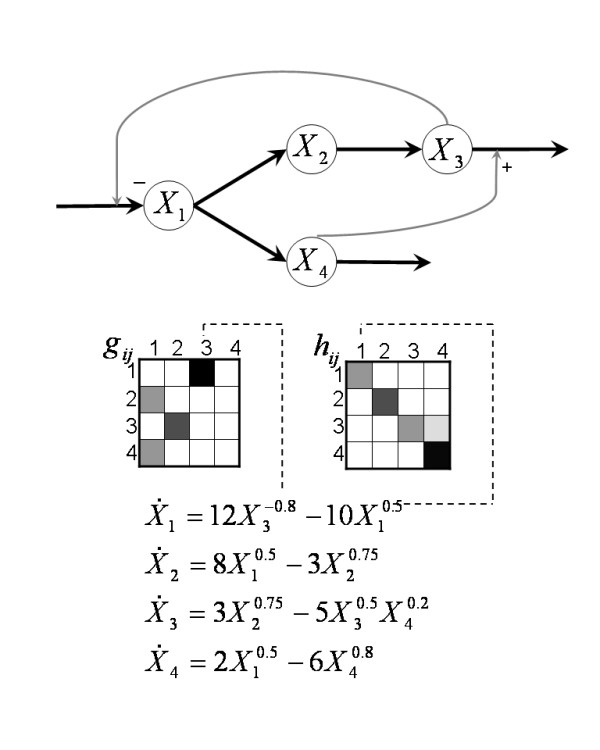
**Topology mapping**. Example of network topology mapping onto kinetic orders in an S-system [17]. The exponents in the equations directly correspond to effects of metabolites on processes (flux arrows) in the pathway diagram. As an example, the flux out of *X*_3 _is affected by *X*_3 _as substrate and by *X*_4 _as activator. Both variables appear in the corresponding term with their respective kinetic orders. The gray-scale in the *g *and *h *matrices reflects the magnitudes of the exponents in the production and degradation terms of the S-system, respectively, with higher values shown in darker hues.

Here, *X*_*i *_represents the concentration of metabolite *i*, *α*_*i *_and *β*_*i *_are non-negative rate constants, and *g*_*ij *_and *h*_*ij *_are real-valued kinetic orders for the production and degradation term, respectively. A considerable amount of information about S-systems can be found in [1-5]. A major advantage of this representation is that it uniquely maps dynamical and topological information onto its parameters; an illustration is given in Figure 1.

Several numerical techniques have been proposed in the literature to tackle the inverse problem of S-system parameterization from time series; most of them use computationally expensive meta-heuristics such as Genetic Algorithms (GA) [6-11], Simulated Annealing (SA) [12], artificial neural networks [13], function approximation [14,15], or global optimization methods [16]. Collectively, these studies have shown that any direct parameter estimations typically face grave problems. Major improvements in efficiency are found when the derivatives at a series of time points are replaced with estimated slopes [4-6] and [17]. This step at once replaces the differential equations with sets of algebraic equations and decouples these sets so that the parameters for each metabolite can be computed separately.

Differing from expensive direct estimation methodologies, alternating regression (AR) [18] was proposed as a fast deterministic method for S-system parameter estimation with low computational cost (see Methods Section). Its superb efficiency is due to the reduction of the nonlinear estimation problem into iterative steps of linear regression. Apparently its only disadvantage is the observation that the method does not converge for some systems, and that necessary and sufficient criteria for convergence are not known. Thus, given a new system and new data, it is *a priori *difficult to predict whether AR will or will not converge. If it converges, it converges very fast.

In this report, we propose a new method, inspired by AR and based on multiple linear regression and sequential quadratic programming (SQP) optimization, to address the S-system parameter identification problem when no information about the network topology is known. The algorithm accounts for the often observed quasi-redundancy among S-system parameters, where errors in kinetic orders can largely be compensated by adjustments in other kinetic orders and rate constants. In contrast to AR, the proposed method operates initially only on one term (production or degradation), whose constant rate (*α *or *β*) and kinetic orders (*g*'s and *h*'s) are optimized completely before the complementary term is estimated. In many cases, the method provides alternative candidate models that fit the time series both in the decoupled and the fully integrated forms.

## Results

### Synthetic time series

The proposed method was tested on synthetic time series generated by reference test models [11,18,19] of 2, 4, and 5 state variables (Equations 2, 3, and 4 respectively). Each system was simulated with different initial concentrations of its variables in order to imitate different biological stimulus-response experiments as described in [18]. All specifications of the simulations with different initial conditions can be found in Additional file 1.

In all three case studies, no knowledge about the pathway was assumed and all parameters were considered freely variable. Even so, the correct network topology was extracted in all cases, with a mean error magnitude of 10^-5 ^for each numerically integrated state variable.

The 2-dimensional system

$$\begin{array}{l} {\dot{X}}_{1}=3X_{2}^{-2}-X_{1}^{0.5}X_{2}\\ {\dot{X}}_{2}=X_{1}^{0.5}X_{2}-X_{2}^{0.5} \end{array}$$

exhibits oscillatory behavior that is challenging for estimation purposes, leading to difficulties of standard algorithms in finding good solutions. The reason is that even small shifts in the oscillation phase between the dynamics of the estimated system and the true target system result in significant cumulative errors. By contrast, the 4-dimensional system

$$\begin{array}{l} {\dot{X}}_{1}=12X_{3}^{-0.8}-10X_{1}^{0.5}\\ {\dot{X}}_{2}=8X_{1}^{0.5}-3X_{2}^{0.75}\\ {\dot{X}}_{3}=3X_{2}^{0.75}-5X_{3}^{0.5}X_{4}^{0.2}\\ {\dot{X}}_{4}=2X_{1}^{0.5}-6X_{4}^{0.8} \end{array}$$

(see Figure 1 for the corresponding pathway) is relatively well behaved and will be used to identify problems that are likely to emerge even for the inference of less complicated dynamic models. The third system (Equation 4) describes an artificial genetic network and has been used as a benchmark [11,18,20] for S-system inference algorithms.

$$\begin{array}{l} {\dot{X}}_{1}=5X_{3}X_{5}^{-1}-10X_{1}^{2}\\ {\dot{X}}_{2}=10X_{1}^{2}-10X_{2}^{2}\\ {\dot{X}}_{3}=10X_{2}^{-1}-10X_{2}^{-1}X_{3}^{2}\\ {\dot{X}}_{4}=8X_{3}^{2}X_{5}^{-1}-10X_{4}^{2}\\ {\dot{X}}_{5}=10X_{4}^{2}-10X_{5}^{2} \end{array}$$

The results of the algorithm on the 2, 4 and 5-dimensional systems, presented in Additional file 1, demonstrate that the proposed method retrieves the correct parameter values for noise-free time series. Three different data sets were created for each test systems (Equations 2, 3 and 4) using different initial conditions in the system's numerical integration (see Additional file 1). These three data sets allowed us to assess the ability of the algorithm to deal with different time series dynamics. Using each data set, we performed 10 trials for each system's variables (*X*_*i*_). The runs differed in the random initial guess for *β *(see *Initial parameter guesses *section for the initialization of the kinetic order values) which was chosen from the range [0.1, 12]. The search space for kinetic orders was limited to a reasonable range of [-2, 3], which is consistent with collective experience in the field (see Chapter 5 in [4]). As an example result, the experiment with the 5-dimensional system performed on the first data set illustrates the success rate of the algorithm: the exact parameter values were found for all variables in all trails except for variable *X*_5 _in one of the trials. The procedure is computationally efficient, requiring 3 minutes to perform 40 optimizations for the 4-dimensional system (10 optimizations for each state variable corresponding to approximately 5 seconds per case), on a personal computer with a 2.00 GHz processor and 1 GB RAM. Thanks to the numerical decoupling, the complexity of the algorithm is of the order O(M*N) where *M *is the number of state variables and *N *is the number of data points used in the optimization. All experiments were performed with 100 data points. For the 5-dimensional system the proposed algorithm found the correct parameter set, overcoming the problematic identification of the kinetic orders *g*_32 _and *h*_32 _of the state variable *X*_3 _presented by most algorithms in the literature. If a stop criterion is defined as a value of 1e-12 for the sum of the squared errors between the slopes of the optimized system and the true slopes, the time required to identify the system parameters for the 5-dimensional system was 23 sec on the machine described above. An experiment with a 10-dimensional system was also performed and the total time consumed was 75 sec (see Additional file 1).

Similar results were achieved with the optimization of the 2-dimensional system. Importantly, the correct parameter set was found, although not with the same regularity as in the 4- and 5-dimensional system optimizations. Issues encountered in finding the correct solutions appeared to be caused by a combination of different features of the system, such as the position of the optimal point within the feasible parameter space, which in the 5-variable case is situated right on the border of the infeasible region within the parameter space (see Figure 1 of the Additional file 1), multiple local minima, as well as the particular choice of initial parameter guesses. These peculiarities of the algorithm and the problem itself lead to different parameter values, although the errors of the decoupled and integrated system are still small (typically about at the order of 1e-5; for instance, see Tables 23, 29 and 30 in the Additional file 1).

The proposed algorithm calculates the initial guesses for the kinetic orders as close to zero as possible, given an initial *β *value (see section *Initial parameter guesses*). However, in this specific case study, near-zero values of the kinetic orders *h*_11 _and *h*_12 _for the constant rate *β*_1 _= 1 fall into the infeasible parameter region, which complicates the parameter optimization. For instance, the smallest feasible value for *h*_12 _is 0.8636. The proposed algorithm overcomes this initial problem by adjusting itself and subsequently returns correct solutions when the system is rescaled in time [21]. This is most easily achieved by multiplying the alphas (*α*_1 _and *α*_2_) and betas (*β*_1 _and *β*_2_) with a positive factor (see example in Additional file 1), which increases the feasible parameter space. This step is, in fact, equivalent to multiplying the slope vector by a positive number. Thanks to the modularity of the decoupled system, this scaling can be performed separately for each state variable without affecting the kinetic order values. Only the values of the rate constants are changed, but they are easily recovered by dividing them by the positive number used for scaling. It was observed that this strategy often, but not always, enhances the algorithmic performance. It appears to improve performance most if the rate constants have small values.

**Figure 2:**
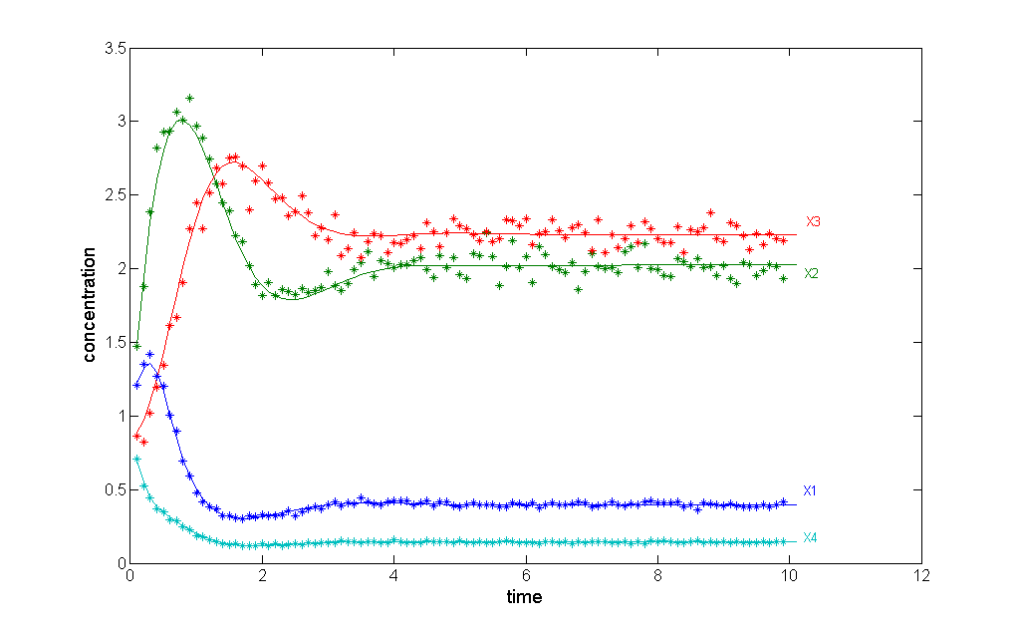
**Noisy time series**. Noisy time series data (symbols) and results of the numerical integration of the estimated model (solid lines; *cf*. Eq. (3)). In spite of slight numerical discrepancies between the estimated parameters and their target values (see Additional file 1), the estimated model accurately predicts the dynamics of the target system, indicating quasi-redundancy [*e.g*., [27] and [25] ] or "sloppiness" [26] among the parameters.

Initially, all experiments were performed with noise-free time series, but in a second set of experiments, we added noise. Because the proposed algorithm uses the decoupled, algebraic form, a signal extraction procedure was employed for the noisy data to provide smooth time series and slopes [22]. The results show that combining the two strategies (smoother and proposed algorithm) generate accurate dynamical responses for the case studies used in this report (Figure 2).

### Error surfaces of decoupled S-systems

**Figure 3:**
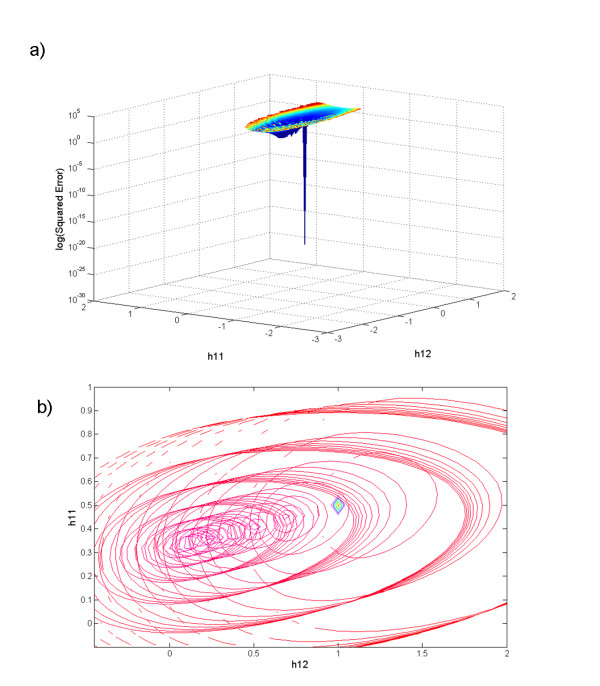
**Error surfaces**. a) Ten error surfaces associated with variable *X*_1 _of the 2-dimensional system were obtained using an exhaustive grid search covering 10 different initial guesses. b) Zooming in shows the composite contour map (level sets) of the error surfaces.

To explore the results of the proposed algorithm visually and to investigate patterns of convergence, we performed a grid search on the parameters of the 2-dimensional system (Equation 2). Specifically, we searched a 100 × 100 grid where each point represented the kinetic orders *h*_11 _and *h*_12 _over the range [-2.5, 2.0]. Correspondingly, 100 time points for *X*_1 _and *X*_2 _and its correspondent slopes *S*_1 _and *S*_2 _were generated by numerical integration of the 2-dimensional system (Equation 2) with *X*_1_(0) = 3 and *X*_2_(0) = 1 as initial conditions. Methods described in a later section were used on time series of *X*_1 _and *X*_2 _to calculate the regression matrix *L*, and for each given initial value of the rate constant *β*_1 _(uniformly spaced over the interval [1,6]) and for each point of the grid, the error surface for the variable *X*_1 _was constructed. The algorithm started with the degradation term $DT_{1}=\beta_{1}X_{1}^{h_{11}}X_{2}^{h_{12}}$ for the first grid point using a given value for *β*_1 _and the time series points for *X*_1 _and *X*_2_. Subsequently, the production vector (*Vp*_1 _= [log(*α*_1_) *g*_11 _*g*_12_]) was obtained from the slope vector *S*_1_, the regression matrix *L*, and the degradation term *DT*_1 _in Equations (7)–(10). Once all parameter values for variable *X*_1 _in the production and degradation vectors were determined, the estimated slopes were calculated (${\hat{S}}_{1}$ = *PT*_1 _- *DT*_1_) and the logarithm of the sum of the squared errors between these slopes and the target solutions was computed as $error=\log\left(\sum {\left(S_{1}-{\hat{S}}_{1}\right)}^{2}\right)$. This process was repeated for all points on the grid such that an error surface resulted for each *β*_1 _value. In this manner, ten surfaces were constructed using different *β *values; they are shown superimposed in Figure 3.

**Figure 4:**
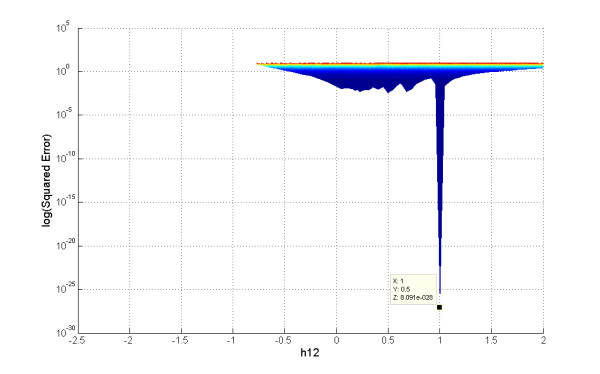
**Multiple minima**. *Z-Y *projection of the error surfaces in Figure 3a. Different minima are found for different *β *values.

The first observation is that most of the search region is not feasible (unfilled *X-Y *space), even though there is *a priori *no hint that solutions in the open range should not converge. It turns out in retrospect that these are regions where the argument of the logarithm on right side of Equation 7 is negative, due to negative slope values. Also worth noting is that for each *β *a similarly shaped surface ("bowl") was found, but that not all surfaces have the same minimal point (Figures 3 and 4). This information will be of critical importance in the discussion of the convergence profile of the proposed method.

The same strategy was applied to noisy time series resulting in a new set of surfaces (data not shown). Gaussian noise with 15% variance was added to the *X*_1 _and *X*_2 _time series and a refined Whitaker's filter [22] was used to smooth the data and estimate slopes.

**Figure 5:**
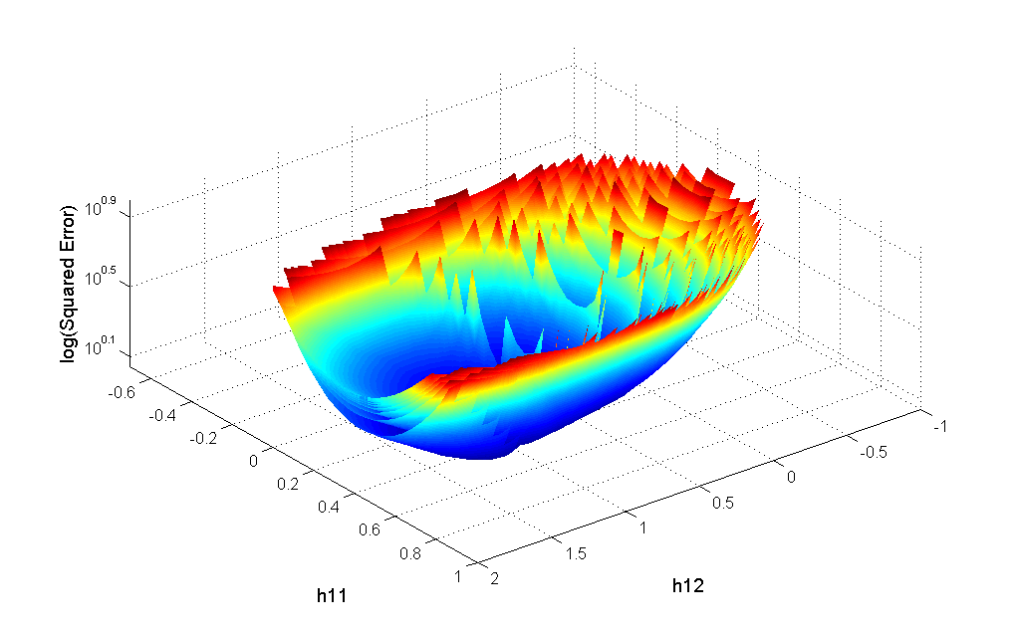
**Error surfaces from noisy time series**. Ten error surfaces of the variable *X*_1 _of the 2-dimensional system obtained from noisy time series after signal extraction and slope estimation.

The error surfaces obtained using noisy data (Figure 5) present the same shapes as seen for the noise-free data except that the error average is higher and points to a different global minimum, which however is essentially indistinguishable in value from the local optima (see Additional file 1 for details).

### Convergence problems

It would be unreasonable to assume that the algorithm converges to the global optimum under all imaginable conditions and initial settings: no estimation algorithm for nonlinear systems can – or should be expected to – measure up to such high a standard. For instance, if the ranges of initial guesses are changed or if the number of initial guesses is reduced, the algorithm may converge to an acceptable local minimum which, however, is not global. This is not surprising, given the complicated nature of the error surface of realistic systems and the fact that nonlinear systems often exhibit almost flat, banana-shaped or ellipsoid valleys in which the minimum is centered [23-27]. At this point, a comprehensive picture of potential obstacles to convergence is not available. One prominent reason for lacking or faulty convergence is that some problems are ill-posed, for instance, because of collinearity between columns of the regression matrix *L*. This situation occurs when two or more metabolites have similar dynamics [25] or when at least one variable is essentially constant and is therefore collinear with the first column of the *L *matrix. In these and some other cases, the regression matrix *L *has a high condition number, which the proposed procedure flags. It might be possible to remedy some of these ill-posed problems with a regularization algorithm for multiple linear regression and through redesigning the algorithm with the regularized solution. It seems advisable in any event to remove model redundancies, for instance by pooling or eliminating collinear variables or merging essentially constant variables with the rate constants of the term.

### Parameter estimation of constrained networks

**Figure 6:**
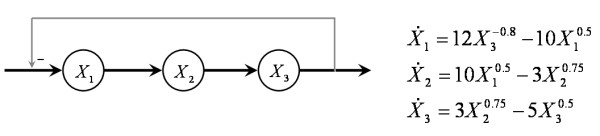
**Linear system topology**. Linear pathway with precursor-product constraints.

The proposed method was extended to address the parameter identification for systems with topological constraints. This extension allows the algorithm to account for precursor-product relationships problems, which mandate that the degradation term of the precursor is equivalent to the production term of the product [28]. Thus, instead of optimizing the parameters for each metabolite separately, a set of terms is optimized simultaneously, consisting of one of the parameter vectors (production or degradation vector) of each metabolite. As an illustrative, simple example, consider a linear pathway with feedback, where we have to account for constraints between the production and degradation terms of subsequent metabolites (Figure 6). Specifically in the example system, the efflux from *X*_1 _is identical to the influx into *X*_2_, and the efflux from *X*_2 _is identical to the influx into *X*_3_. Consequently, the degradation term of *X*_1 _is exactly the same as the production term of *X*_2_, and the degradation term of *X*_2 _must be the same as the production term of *X*_3_. The amendment of the proposed method toward simultaneous estimation readily satisfies these types of constraints.

The extended algorithm was applied to the 3-dimensional linear pathway system in Figure 6, and some of the results are shown in Additional file 1. The algorithm found the correct parameter set, and all 10 optimizations, in which the algorithm now performs a single, combined optimization for all variables simultaneously, thereby accounting for constraints, were completed in 37 sec on a 2.00 GHz processor with 1 GB RAM.

### Graphical user interface

**Figure 7:**
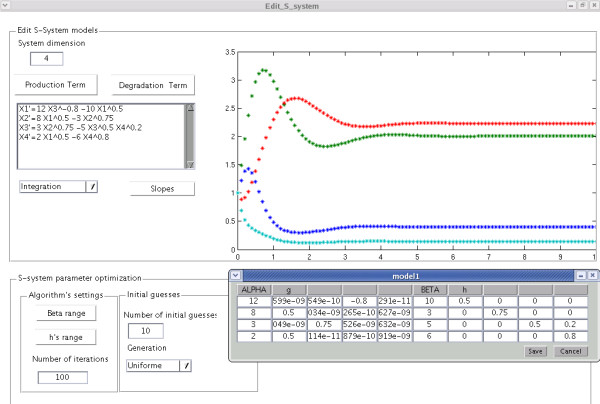
**Software application**. Snapshot of the graphical user interface provided as a free stand-alone application.

An open source MATLAB toolbox and a stand-alone compiled Graphical User Interface (GUI) application were developed as an exploratory tool (see Section *Availability and requirements*). The application was developed as a modular extension of our previous work and constitutes a critical component within our long-term effort of advancing a data processing pipeline for S-system estimation from metabolomic time series [13,22]. A snapshot of the GUI is shown in Figure 7. All computational results and graphics described in this report can be reproduced using this application.

## Discussion

There are many reasons why it may be desirable to reverse engineer a biological network without making assumptions about the underlying processes. The most obvious reason is that no reliable information may be available about the processes. Another situation occurs when several network topologies are *a priori *possible and the reverse approach is employed to prioritize alternative hypotheses. The algorithm proposed here is an extension of Alternating Regression (AR; [18]) that in many cases shows improved convergence behavior.

The proposed algorithm was exhaustively tested on diverse time series (see Text above and Additional File 1). In all of these tests, the convergence followed the same pattern: the error slowly decreased during the first few iterations and then suddenly dropped to a significant lower plateau, from where it gradually decreased again. This pattern repeated until one of the stop conditions (maximal number of iterations, minimal gradient value or minimal cost function value) was reached. The error drop points matched with significant changes in the beta gradient and appear to correspond to transitions to a "bowl" with a lower error surface (*cf*. Figures 3 and 5). As shown in Figures 3b and 5, most "bowls" have different minimal points, corresponding to good, yet local minima. Because the proposed algorithm is computationally very efficient, it allows the exploration of the parameter space in a reasonable amount of time (seconds to minutes). Such an exploration with new initial *β *values is recommended, if very precise solutions or alternative parameter sets are needed. Because alternative parameter combinations may correspond to different topological and regulatory structures [4], estimations with different initial values in fact constitute explorations of the structure and functionality of the biological space in which the pathway operates.

## Conclusion

S-systems present a unique balance between proven biological relevance and validity on one hand, and mathematical convenience and tractability on the other. For this reason, the recent years have seen numerous methods for matching S-system models to measured biological time series data. In the relatively simpler scenario of this type, the topology and regulatory structure of the biological system is known, and the extraction of information from the data constitutes a parameter estimation task. In the more difficult situation, at least some of the structure is unknown, and in the extreme situation no information about the topology of the interactions between variables is available. In this article we propose a new algorithm that efficaciously identifies the correct topology of a system from time series. The only true assumptions made are that all important variables are accounted for and that the S-system model is capable of modeling the data. The first assumption is presently unavoidable, at least in the generality presented above. The second assumption has been found to be true in very many cases, as a rich body of publications on S-systems demonstrates. The proposed algorithm was conceived as a critical piece of an emerging data processing "pipeline" that will eventually accept time series and other data characterizing biological pathways and more or less automatically propose topological and regulatory structures that are consistent with the input data. This algorithm will be a valuable tool for analysis and hypothesis generation in systems biology.

## Methods

### Eigenvector optimization

The proposed method was inspired by Alternating Regression (AR [18]) and is based on the substitution of differentials with estimated slopes [4,5,17] and the minimization of the differences between two vectors obtained from multiple linear regression equations. In contrast to AR, the new algorithm estimates one term per equation with high accuracy and computes the other term through linear regression ensuring that the new term will fall into the feasible space. Specifically, the task is initially posed in relation to one of the two terms of an S-system equation with *M *species (*e.g*., metabolites), either the production term vector $PT_{i}(t_{n})=\alpha_{i}\prod_{j=1}^{M}X_{j}{\left(t_{n}\right)}^{g_{ij}}$ or the degradation term vector $DT_{i}(t_{n})=\beta_{i}\prod_{j=1}^{M}X_{j}{\left(t_{n}\right)}^{h_{ij}}$, which are both defined for each metabolite *i *at a series of *N *time points *t*_*n*_. Let *S*_*i*_(*t*_*n*_) denote the estimated slope of metabolite *i *at time *t*_*n*_. In simplified notation, *S*_*i*_(*t*_*n*_) is given by

*S*_*i*_(*t*_*n*_) = *PT*_*i*_(*t*_*n*_) - *DT*_*i*_(*t*_*n*_), *n *= 1, 2, ⋯,*N*

Because *PT*_*i *_must be positive, Equation 6 can be rewritten as

log(*PT*_*i*_) = log(*S*_*i *_+ *DT*_*i*_),

or in matrix form as

*L*·*Vp*_*i *_= *y*_*i*_,

where the production parameter vector is given as *V*_*pi *_= [log *α*_*i *_*g*_*i*1 _*g*_*i*2 _⋯ *g*_*iM*_], *y*_*i *_= log(*S*_*i *_+ *DT*_*i*_), and the regression matrix *L *is

$$L=\left[\begin{array}{cccccc} 1 & \log(X_{1}(t_{1})) & \cdots & \log(X_{i}(t_{1})) & \cdots & \log(X_{M}(t_{1}))\\ 1 & \log(X_{1}(t_{2})) & \cdots & \log(X_{i}(t_{2})) & \cdots & \log(X_{M}(t_{2}))\\ \vdots & \vdots & \cdots & \vdots & \cdots & \vdots \\ 1 & \log(X_{1}(t_{N})) & \cdots & \log(X_{i}(t_{N})) & \cdots & \log(X_{M}(t_{N})) \end{array}\right].$$

As is standard with multiple linear regression models, the production parameter vector *Vp*_*i *_can be obtained as

*Vp*_*i *_= (*L*^*T*^*L*)^-1 ^*L*^*T*^*y*_*i*_,

as long as the inverse exists. Substituting this result in Equation 8 directly yields

*L*(*L*^*T*^*L*)^-1 ^*L*^*T*^*y*_*i *_= *y*_*i*_.

Recall that vector *y*_*i *_is a function of the degradation parameters (*β *_*i *_and *h*_*ij*_), which thus must satisfy Equation 11. Specifically, *y*_*i *_must be an eigenvector of the matrix *W *= *L*(*L*^*T*^*L*)^-1^*L*^*T *^with an eigenvalue equalling 1.

We used several standard algorithms to calculate the eigenvector of the matrix *W *directly, but none of them returned a satisfactory result. The presumed reason is that any vector which belongs to the eigenspace of *W *corresponding to eigenvalue 1 satisfies the Equation 11. We therefore forced the eigenvector *y*_*i *_to be in the form log(*S*_*i*_+*DT*_*i*_) and reformulated the task as a minimization problem for the logarithm of the squared residuals between the right and left side hands in Equation 11 and defined this problem in matrix form with the cost function

$$F=\log\left({\left({\hat{y}}_{i}-y_{i}\right)}^{T}\left({\hat{y}}_{i}-y_{i}\right)\right),$$

where ${\hat{y}}_{i}=Wy_{i}$. The gradients of this function with respect to the degradation parameters are given by Equations 13 and 14:

$$\frac{\partial F}{\partial \beta_{i}}=\frac{2}{\phi }{\left[\left(W-I\right)\left(\left(\prod_{j=1}^{M}X_{j}^{h_{ij}}\right)\circ {\left(S_{i}+DT_{i}\right)}^{\circ -1}\right)\right]}^{T}\left[\left(W-I\right)\log\left(S_{i}+DT_{i}\right)\right],$$

$$\frac{\partial F}{\partial h_{ij}}=\frac{2}{\phi }{\left[\left(W-I\right)\left(\left(\beta_{i}\prod_{j=1}^{M}X_{j}^{h_{ij}}\circ \log\left(X_{j}\right)\right)\circ {\left(S_{i}+DT_{i}\right)}^{\circ -1}\right)\right]}^{T}\left[\left(W-I\right)\log\left(S_{i}+DT_{i}\right)\right].$$

Here, the symbol *ο *represents the Hadamard product between vectors [29] and *φ *is the logarithm of the argument of the right hand side of the Equation 12. Analogous gradient equations are obtained for the production terms. The algorithm avoids unfeasible solutions by satisfying the feasibility constraints

$$S_{i}(t_{n})+\beta_{i}\prod_{j=1}^{M}X_{j}{\left(t_{n}\right)}^{h_{ij}}>0,n=1,2,\cdots ,N.$$

We used the **fmincon **routine in MATLAB^® ^(MathWorks) with built-in Sequential Quadratic Programming to execute the cost function constrained minimization.

### Initial parameters guesses

**Figure 8:**
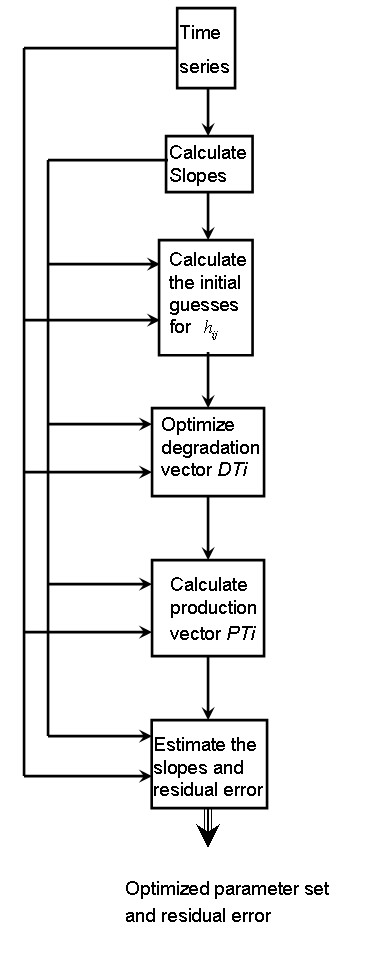
**Flowchart**. Flowchart of the proposed algorithm. To perform the optimization process, the algorithm requires only the time series set and an initial *β *value as input.

Like all numerical optimization algorithms, the proposed method requires initial guesses. Satisfying the constraints in Equation 15, the proposed algorithm calculates initial guesses for the kinetic order *h*_*ij*_, given a user-supplied value *β*_*i*_; specifically, *h*_*ij *_and a small buffer value *ε *are chosen such that

$$\beta_{i}\prod_{j=1}^{M}X_{j}^{h_{ij}}=\varepsilon -S_{i}^{-},$$

where $S_{i}^{-}$ represents all negative slope values from the time series of *X*_i_. A simple linear regression step in logarithmic space thus suffices to determine admissible initial guesses for the kinetic orders *h*_*ij*_. In this fashion, for a given *β*_*i*_, small values of kinetic orders *h*_*ij *_are provided to the optimization algorithm. As a technical note, it is easier to keep a null parameter value than to bring it to zero during the optimization. If the slope vector contains no negative values, the procedure is performed without *ε*. A flowchart of the proposed algorithm is shown in Figure 8.

### Refining solutions

Differently parameterized S-systems can exhibit quite similar temporal dynamics. This behavior is due the fact that S-systems are composed of production and degradation terms that may compensate for each other through different kinetic orders and constant rates that ultimately produce very similar time courses. As one consequence, it is quite common that optimization schemes identify non-zero values for parameters that should in truth be zero. Moreover, it is unlikely that any algorithm based on gradients will obtain parameters values exactly equal to zero. For these reasons, our algorithm automatically checks parameter values and forces kinetics orders below a quite arbitrary threshold of (0.009) to be zero; a new optimization process is the initiated in which the parameter is constrained to be zero.

### Extension to constrained topologies

To address linear pathway sections, constraints are imposed in accordance with the structure of the system when the parameter optimization is performed. For instance, for the linear system with precursor-product relationships (Figure 6), the optimization is performed with the degradation term of the precursor metabolite forced to be equal to the production terms of the product. In such a case, the Equation 11 is formulated for each state variable

$$\begin{array}{c} Wy_{1}(\beta_{1},h_{1j},S_{1})=y_{1}(\beta_{1},h_{1j},S_{1})\\ Wy_{2}(\alpha_{2},g_{2j},S_{2})=y_{2}(\alpha_{2},g_{2j},S_{2})\\ \vdots \\ Wy_{M}(\alpha_{M},g_{Mj},S_{M})=y_{M}(\alpha_{M},g_{Mj},S_{M}) \end{array},$$

and the sum of the equations returns the eigenvector problem

$$W\left(\sum_{i=1}^{M}y_{i}\right)=\sum_{i=1}^{M}y_{i}.$$

A cost function similar to Equation 12 can be formulated using the Equation 18, and the same optimization procedure is used. To force flux conservation, the following constraints were imposed on the optimization algorithm

$$\begin{array}{l} \beta_{1}=\alpha_{2}\\ h_{1j}=g_{2j},j=1,2,..,M \end{array}$$

to impose

*DT*_1 _= *PT*_2_,

and the degradation term of *X*_2 _was forced to be equal the production term of *X*_3_

$$\begin{array}{l} PT_{3}=DT_{2}\\ \alpha_{3}\prod_{j=1}^{M}X_{j}^{g_{3j}}=PT_{2}-S_{2} \end{array}.$$

Applying logarithms on both sides of the Equation 21 and solving the equation by multiple linear regression, the final constraints are found as

$$\alpha_{3}=\prod_{n=1}^{N}{\left(PT_{2}(t_{n})-S_{2}(t_{n})\right)}^{C_{1n}},$$

and

$$g_{3j}=\sum_{n=1}^{N}C_{j+1,n}\log\left(PT_{2}(t_{n})-S_{2}(t_{n})\right),j=1,2,..,M,$$

where *C *= (*L*^*T*^*L*)^-1^*L*^*T*^. The constraints can be rewritten in a general form as

$$\alpha_{M}=\prod_{n=1}^{N}{\left(PT_{M-1}(t_{n})-S_{M-1}(t_{n})\right)}^{C_{1n}},$$

and

$$g_{Mj}=\sum_{n=1}^{N}C_{j+1,n}\log\left(PT_{M-1}(t_{n})-S_{M-1}(t_{n})\right),j=2,..,M.$$

Analogous optimization routines were used for other constraints.

## Availability and requirements

The implementation of the algorithm described in this report is made publicly (GNU GPL) available with open source as Matlab m-code (MathWorks Inc) at . For the convenience of those without a Mathworks license we have also compiled the code as a stand-alone application made publicly available at the same site, or as a module ("Signal Extraction Toolbox") of the code distribution infrastructure of the Bioinformatics Station resource .

## Competing interests

The author(s) declare that they have no competing interests.

## Authors' contributions

MV conceived the core methods of eigenvector optimization. I-Chun Chou spearheaded Alternating Regression and tested the eigenvector method.

SV participated in the analysis and systematization of the method.

ATV supported the development of the proposed algorithm with funds and designed the computational tests.

EOV initiated the field of network identification with S-systems and supervised activities leading to this paper.

JSA conceived the ideas of automating the identification of S-systems and creating a model pipeline.

All authors contributed to in the preparation of the manuscript.

## Supplementary Material

Additional file 1 Supplementary material. Click here for file
