# Supplementary material for: Parameter optimization in S-system models
Source: BMC Syst Biol. 2008 Apr 16;2:35. doi: 10.1186/1752-0509-2-35 (PMC2333970; doi:10.1186/1752-0509-2-35)
Supplement: Additional file 1 — Supplementary material. [file 1752-0509-2-35-S1.doc]

**Supplementary material to**

## Parameter optimization of S-systems models

**Marco Vilela, I-Chun Chou, Susana Vinga, Ana Tereza R. Vasconcelos, Eberhard O. Voit and Jonas S. Almeida**

In this additional file, we present some results obtained using the proposed algorithm. All experiments were performed with the systems presented in the main manuscript, namely the 2-dimensional system (Equation 1) [1]

, (1)

the 4-dimensional system (Equation 2) [2]

, (2)

and the 5-dimensional system (Equation 3) [3]

. (3)

Time rescaled versions of the 2- and 4-dimensional systems (Equations (4) and (5)) are also used to demonstrate the efficacy of the proposed method:

, (4)

and

. (5)

In order to test the robustness of the algorithm, we also performed experiments in a 10-dimensional system (Equation 6).

(6)

This system represents a genetic network partially removed from [4].

## Error surfaces

In order to visually explore the results of the proposed algorithm and clarify the pattern of convergence, several error surfaces are presented in this supplementary material, all resulting from experiments with the 2- and 4-dimensional systems (Equations 1 and 2). The surfaces were built with the same procedure described in the section *Error surfaces of decoupled S-system* in the main manuscript.

Figure 1: Error surface (for $\beta_{1}$=1) of the state variable *X*_1_ of the 2-dimensional system (Equation 1). The optimal point is positioned close to the border of the feasible parameter space, making difficult the optimization.

Figure 2: Z-Y projection of the error surfaces shown in the Figure 5 of the main manuscript obtained with noisy time series. The optimal point (labeled) is not conserved from the noise-free error surfaces, but it is essentially indistinguishable from local minimum.

Figure 3: Error surfaces (for $\beta_{1}$=10 and $\beta_{1}$=12) of the state variable *X*_1_ of the 4-dimensional system (Equation 2). Only the kinetic orders *h*_11_ and *h*_12_ were screened (*h*_13_ and *h*_14_ were set to zero).

Figure 4: Error surfaces (for $\beta_{2}$=2 and $\beta_{2}$=3) of the state variable *X*_2_ of the 4-dimensional system (Equation 2). Only the kinetic orders *h*_21_ and *h*_22_ were screened (*h*_23_ and *h*_24_ were set to zero).

Figure 5: Error surfaces (for $\beta_{3}$=5 and $\beta_{3}$=7) of the state variable *X*_3_ of the 4-dimensional system (Equation 2). Only the kinetic orders *h*_33_ and *h*_34_ were screened (*h*_31_ and *h*_32_ were set to zero).

Figure 6: Error surfaces (for $\beta_{4}$=4 and $\beta_{4}$=6) of the state variable *X*_4_ of the 4-dimensional system (Equation 2). Only the kinetic orders *h*_43_ and *h*_44_ were screened (*h*_41_ and *h*_42_ were set to zero).

## Numeric experiments

To perform the experiments, different initial conditions for the systems variables were chosen (Table 1, 2 and 3) to generate time series by numerical integration. For each of these conditions, 10 runs were performed for each system’s variable. In all result tables, the sums of squared error in relation with the decoupled and numerically integrated system are present as Error1 and Error2 respectively. All data sets were generated with the software PLAS [5].

| Initial Condition | $X_{1}\left(0\right)$ | $X_{2}\left(0\right)$ | $X_{3}\left(0\right)$ | $X_{4}\left(0\right)$ |
| --- | --- | --- | --- | --- |
| 1 | 1.0 | 1.0 | 1.0 | 1.0 |
| 2 | 1.0 | 3.0 | 1.3 | 1.3 |
| 3 | 1.5 | 0.5 | 0.5 | 1.5 |

Table 1 – Initial conditions for integration of the 4-dimensional system

| Initial Condition | $X_{1}\left(0\right)$ | $X_{2}\left(0\right)$ | $X_{3}\left(0\right)$ | $X_{4}\left(0\right)$ | $X_{5}\left(0\right)$ |
| --- | --- | --- | --- | --- | --- |
| 1 | 0.10 | 0.70 | 0.70 | 0.16 | 0.18 |
| 2 | 0.70 | 0.12 | 0.14 | 0.16 | 0.18 |
| 3 | 0.70 | 0.70 | 0.14 | 0.16 | 0.70 |

Table 2 – Initial conditions for integration of the 5-dimensional system

| Initial Condition | $X_{1}\left(0\right)$ | $X_{2}\left(0\right)$ |
| --- | --- | --- |
| 1 | 3.0 | 1.0 |
| 2 | 1.5 | 1.5 |
| 3 | 0.75 | 1.5 |

Table 3 – Initial conditions for integration of the 2-dimensional system

## 4-Dimensional system results – noise-free time series

**Data set 1**

Tables 4-7 show the parameters found with the proposed algorithm for the 4-dimensional system (Equation 2) using the first set of initial values of the Table 1. The time series used in this case study for all datasets were obtained by numerical integration of the 4-dimensional system in the interval [0,10] with 0.1 sampling interval.

| run | $\alpha_{1}$ | $g_{11}$ | $g_{12}$ | $g_{13}$ | $g_{14}$ | $\beta_{1}$ | $h_{11}$ | $h_{12}$ | $h_{13}$ | $h_{14}$ | Error1 | Error2 |
| --- | --- | --- | --- | --- | --- | --- | --- | --- | --- | --- | --- | --- |
| 1 | 12.00 | 0.00 | 0.00 | -0.80 | 0.00 | 10.00 | 0.50 | 0.00 | 0.00 | 0.00 | 1.75483E-20 | 1.33873E-05 |
| 2 | 12.00 | 0.00 | 0.00 | -0.80 | 0.00 | 10.00 | 0.50 | 0.00 | 0.00 | 0.00 | 1.71974E-19 | 1.21796E-05 |
| 3 | 12.00 | 0.00 | 0.00 | -0.80 | 0.00 | 10.00 | 0.50 | 0.00 | 0.00 | 0.00 | 5.68827E-19 | 1.21338E-05 |
| 4 | 12.00 | 0.00 | 0.00 | -0.80 | 0.00 | 10.00 | 0.50 | 0.00 | 0.00 | 0.00 | 1.71382E-19 | 1.31119E-05 |
| 5 | 12.00 | 0.00 | 0.00 | -0.80 | 0.00 | 10.00 | 0.50 | 0.00 | 0.00 | 0.00 | 9.24261E-19 | 1.46669E-05 |
| 6 | 12.00 | 0.00 | 0.00 | -0.80 | 0.00 | 10.00 | 0.50 | 0.00 | 0.00 | 0.00 | 4.38633E-19 | 1.22694E-05 |
| 7 | 12.00 | 0.00 | 0.00 | -0.80 | 0.00 | 10.00 | 0.50 | 0.00 | 0.00 | 0.00 | 5.29743E-19 | 1.3364E-05 |
| 8 | 12.00 | 0.00 | 0.00 | -0.80 | 0.00 | 10.00 | 0.50 | 0.00 | 0.00 | 0.00 | 3.03162E-20 | 1.32749E-05 |
| 9 | 12.00 | 0.00 | 0.00 | -0.80 | 0.00 | 10.00 | 0.50 | 0.00 | 0.00 | 0.00 | 6.26499E-19 | 1.3495E-05 |
| 10 | 12.00 | 0.00 | 0.00 | -0.80 | 0.00 | 10.00 | 0.50 | 0.00 | 0.00 | 0.00 | 1.62522E-20 | 1.21132E-05 |

Table 4 – Result of the 10 runs for the variable *X*_1_ of the 4-dimensional system with beta initial guesses randomly distributed in the range [1, 12].

| run | $\alpha_{2}$ | $g_{21}$ | $g_{22}$ | $g_{23}$ | $g_{24}$ | $\beta_{2}$ | $h_{21}$ | $h_{22}$ | $h_{23}$ | $h_{24}$ | Error1 | Error2 |
| --- | --- | --- | --- | --- | --- | --- | --- | --- | --- | --- | --- | --- |
| 1 | 8.00 | 0.50 | 0.00 | 0.00 | 0.00 | 3.00 | 0.00 | 0.75 | 0.00 | 0.00 | 4.73294E-20 | 9.0079E-05 |
| 2 | 8.00 | 0.50 | 0.00 | 0.00 | 0.00 | 3.00 | 0.00 | 0.75 | 0.00 | 0.00 | 1.94596E-19 | 8.7003E-05 |
| 3 | 8.00 | 0.50 | 0.00 | 0.00 | 0.00 | 3.00 | 0.00 | 0.75 | 0.00 | 0.00 | 2.42293E-18 | 8.6766E-05 |
| 4 | 14.10 | 0.35 | 0.19 | -0.03 | 0.02 | 9.10 | 0.11 | 0.53 | -0.03 | 0.02 | 3.74444E-05 | 0.00010221 |
| 5 | 16.18 | 0.33 | 0.21 | -0.02 | 0.02 | 11.18 | 0.13 | 0.50 | -0.03 | 0.02 | 4.24392E-05 | 0.00010673 |
| 6 | 8.00 | 0.50 | 0.00 | 0.00 | 0.00 | 3.00 | 0.00 | 0.75 | 0.00 | 0.00 | 2.44827E-20 | 8.7397E-05 |
| 7 | 16.47 | 0.33 | 0.22 | -0.02 | 0.02 | 11.47 | 0.14 | 0.50 | -0.02 | 0.02 | 4.62284E-05 | 0.00010031 |
| 8 | 13.40 | 0.36 | 0.18 | -0.01 | 0.01 | 8.40 | 0.12 | 0.54 | -0.01 | 0.02 | 3.76855E-05 | 9.9757E-05 |
| 9 | 15.86 | 0.33 | 0.21 | -0.02 | 0.02 | 10.86 | 0.13 | 0.51 | -0.02 | 0.02 | 4.29122E-05 | 0.00010555 |
| 10 | 8.00 | 0.50 | 0.00 | 0.00 | 0.00 | 3.00 | 0.00 | 0.75 | 0.00 | 0.00 | 5.2642E-19 | 8.6676E-05 |

Table 5 – Result of the 10 runs for the variable *X*_2_ of the 4-dimensional system with beta initial guesses randomly distributed in the range [1, 12].

| run | $\alpha_{3}$ | $g_{31}$ | $g_{32}$ | $g_{33}$ | $g_{34}$ | $\beta_{3}$ | $h_{31}$ | $h_{32}$ | $h_{33}$ | $h_{34}$ | Error1 | Error2 |
| --- | --- | --- | --- | --- | --- | --- | --- | --- | --- | --- | --- | --- |
| 1 | 3.00 | 0.00 | 0.75 | 0.00 | 0.00 | 5.00 | 0.00 | 0.00 | 0.50 | 0.20 | 2.43701E-18 | 7.6974E-05 |
| 2 | 8.63 | 0.02 | 0.53 | 0.19 | 0.07 | 10.63 | 0.03 | 0.22 | 0.40 | 0.15 | 2.20633E-06 | 7.4771E-05 |
| 3 | 10.00 | 0.02 | 0.51 | 0.21 | 0.07 | 12.00 | 0.03 | 0.24 | 0.39 | 0.14 | 2.44862E-06 | 7.467E-05 |
| 4 | 9.99 | 0.02 | 0.51 | 0.21 | 0.07 | 11.99 | 0.03 | 0.24 | 0.39 | 0.14 | 2.42734E-06 | 8.1619E-05 |
| 5 | 3.00 | 0.00 | 0.75 | 0.00 | 0.00 | 5.00 | 0.00 | 0.00 | 0.50 | 0.20 | 1.06429E-18 | 8.7564E-05 |
| 6 | 7.74 | 0.02 | 0.54 | 0.18 | 0.06 | 9.74 | 0.03 | 0.21 | 0.41 | 0.15 | 2.06481E-06 | 7.5389E-05 |
| 7 | 8.17 | 0.02 | 0.53 | 0.18 | 0.06 | 10.17 | 0.03 | 0.21 | 0.40 | 0.15 | 2.1591E-06 | 8.2248E-05 |
| 8 | 7.28 | 0.02 | 0.55 | 0.17 | 0.06 | 9.28 | 0.02 | 0.20 | 0.41 | 0.15 | 1.92141E-06 | 8.0877E-05 |
| 9 | 10.00 | 0.02 | 0.51 | 0.21 | 0.07 | 12.00 | 0.03 | 0.24 | 0.39 | 0.14 | 2.47521E-06 | 8.7686E-05 |
| 10 | 10.00 | 0.02 | 0.51 | 0.21 | 0.07 | 12.00 | 0.03 | 0.24 | 0.39 | 0.14 | 2.41783E-06 | 7.4469E-05 |

Table 6 – Result of the 10 runs for the variable *X*_3_ of the 4-dimensional system with beta initial guesses randomly distributed in the range [1, 12].

| run | $\alpha_{4}$ | $g_{41}$ | $g_{42}$ | $g_{43}$ | $g_{44}$ | $\beta_{4}$ | $h_{41}$ | $h_{42}$ | $h_{43}$ | $h_{44}$ | Error1 | Error2 |
| --- | --- | --- | --- | --- | --- | --- | --- | --- | --- | --- | --- | --- |
| 1 | 8.00 | 0.36 | -0.04 | 0.06 | 0.26 | 12.00 | 0.18 | -0.05 | 0.06 | 0.56 | 8.19133E-08 | 1.3953E-06 |
| 2 | 8.00 | 0.36 | -0.04 | 0.06 | 0.26 | 12.00 | 0.18 | -0.05 | 0.06 | 0.56 | 8.10149E-08 | 1.4304E-06 |
| 3 | 8.00 | 0.36 | -0.04 | 0.06 | 0.26 | 12.00 | 0.18 | -0.05 | 0.06 | 0.56 | 8.16504E-08 | 1.4308E-06 |
| 4 | 8.00 | 0.36 | -0.04 | 0.06 | 0.26 | 12.00 | 0.18 | -0.04 | 0.06 | 0.56 | 7.26348E-08 | 1.4368E-06 |
| 5 | 2.00 | 0.50 | 0.00 | 0.00 | 0.00 | 6.00 | 0.00 | 0.00 | 0.00 | 0.80 | 1.14483E-19 | 1.3818E-06 |
| 6 | 8.00 | 0.36 | -0.04 | 0.06 | 0.26 | 12.00 | 0.18 | -0.05 | 0.06 | 0.56 | 7.88685E-08 | 1.4308E-06 |
| 7 | 2.00 | 0.50 | 0.00 | 0.00 | 0.00 | 6.00 | 0.00 | 0.00 | 0.00 | 0.80 | 1.97709E-19 | 1.3985E-06 |
| 8 | 7.65 | 0.37 | -0.04 | 0.06 | 0.26 | 11.65 | 0.18 | -0.04 | 0.06 | 0.56 | 8.21441E-08 | 1.4234E-06 |
| 9 | 7.83 | 0.36 | -0.04 | 0.06 | 0.26 | 11.83 | 0.18 | -0.04 | 0.06 | 0.56 | 7.9069E-08 | 1.4524E-06 |
| 10 | 8.00 | 0.36 | -0.04 | 0.06 | 0.26 | 12.00 | 0.18 | -0.05 | 0.06 | 0.56 | 8.28791E-08 | 1.4294E-06 |

Table 7 – Result of the 10 runs for the variable *X*_4_ of the 4-dimensional system with beta initial guesses randomly distributed in the range [1, 12].

**Data set 2**

Tables 8-11 show the parameters found with the proposed algorithm for the 4-dimensional system (Equation 2) using the second set of initial values of the Table 1.

| run | $\alpha_{1}$ | $g_{11}$ | $g_{12}$ | $g_{13}$ | $g_{14}$ | $\beta_{1}$ | $h_{11}$ | $h_{12}$ | $h_{13}$ | $h_{14}$ | Error1 | Error2 |
| --- | --- | --- | --- | --- | --- | --- | --- | --- | --- | --- | --- | --- |
| 1 | 12.00 | 0.00 | 0.00 | -0.80 | 0.00 | 10.00 | 0.50 | 0.00 | 0.00 | 0.00 | 6.392E-19 | 1.545E-05 |
| 2 | 12.00 | 0.00 | 0.00 | -0.80 | 0.00 | 10.00 | 0.50 | 0.00 | 0.00 | 0.00 | 8.004E-20 | 2.218E-05 |
| 3 | 12.00 | 0.00 | 0.00 | -0.80 | 0.00 | 10.00 | 0.50 | 0.00 | 0.00 | 0.00 | 3.056E-20 | 1.323E-05 |
| 4 | 12.00 | 0.00 | 0.00 | -0.80 | 0.00 | 10.00 | 0.50 | 0.00 | 0.00 | 0.00 | 4.199E-19 | 3.055E-05 |
| 5 | 12.00 | 0.00 | 0.00 | -0.80 | 0.00 | 10.00 | 0.50 | 0.00 | 0.00 | 0.00 | 2.521E-19 | 1.506E-05 |
| 6 | 12.00 | 0.00 | 0.00 | -0.80 | 0.00 | 10.00 | 0.50 | 0.00 | 0.00 | 0.00 | 1.854E-20 | 3.049E-05 |
| 7 | 12.00 | 0.00 | 0.00 | -0.80 | 0.00 | 10.00 | 0.50 | 0.00 | 0.00 | 0.00 | 1.89E-18 | 2.847E-05 |
| 8 | 12.00 | 0.00 | 0.00 | -0.80 | 0.00 | 10.00 | 0.50 | 0.00 | 0.00 | 0.00 | 7.85E-20 | 1.504E-05 |
| 9 | 12.00 | 0.00 | 0.00 | -0.80 | 0.00 | 10.00 | 0.50 | 0.00 | 0.00 | 0.00 | 1.304E-19 | 2.109E-05 |
| 10 | 12.67 | 2.07 | 1.72 | -0.86 | -0.96 | 12.00 | 2.00 | 1.81 | -1.00 | -0.98 | 0.0001282 | 12.067289 |

Table 8 – Result of the 10 runs for the variable *X*_1_ of the 4-dimensional system with beta initial guesses randomly distributed in the range [1, 12].

| run | $\alpha_{2}$ | $g_{21}$ | $g_{22}$ | $g_{23}$ | $g_{24}$ | $\beta_{2}$ | $h_{21}$ | $h_{22}$ | $h_{23}$ | $h_{24}$ | Error1 | Error2 |
| --- | --- | --- | --- | --- | --- | --- | --- | --- | --- | --- | --- | --- |
| 1 | 16.81 | 0.31 | 0.26 | -0.04 | 0.00 | 12.00 | 0.14 | 0.52 | -0.04 | 0.00 | 2.08874E-08 | 6.8394E-05 |
| 2 | 14.95 | 0.33 | 0.24 | -0.03 | 0.00 | 10.14 | 0.13 | 0.54 | -0.03 | 0.00 | 1.91115E-08 | 0.00011427 |
| 3 | 16.81 | 0.31 | 0.26 | -0.04 | 0.00 | 12.00 | 0.14 | 0.52 | -0.04 | 0.00 | 2.09348E-08 | 5.6501E-05 |
| 4 | 15.87 | 0.32 | 0.25 | -0.03 | 0.00 | 11.06 | 0.14 | 0.53 | -0.03 | 0.00 | 2.01867E-08 | 0.00020999 |
| 5 | 16.81 | 0.31 | 0.26 | -0.04 | 0.00 | 12.00 | 0.14 | 0.52 | -0.04 | 0.00 | 2.08498E-08 | 6.5493E-05 |
| 6 | 16.81 | 0.31 | 0.26 | -0.04 | 0.00 | 12.00 | 0.14 | 0.52 | -0.04 | 0.00 | 2.09342E-08 | 0.0002029 |
| 7 | 8.00 | 0.50 | 0.00 | 0.00 | 0.00 | 3.00 | 0.00 | 0.75 | 0.00 | 0.00 | 2.73208E-19 | 0.00018978 |
| 8 | 16.81 | 0.31 | 0.26 | -0.04 | 0.00 | 12.00 | 0.14 | 0.52 | -0.04 | 0.00 | 2.0928E-08 | 6.5644E-05 |
| 9 | 15.32 | 0.33 | 0.25 | -0.03 | 0.00 | 10.51 | 0.13 | 0.53 | -0.03 | 0.00 | 1.95157E-08 | 0.00013272 |
| 10 | 16.81 | 0.31 | 0.26 | -0.04 | 0.00 | 12.00 | 0.14 | 0.52 | -0.04 | 0.00 | 2.09177E-08 | 255.988849 |

Table 9 – Result of the 10 runs for the variable *X*_2_ of the 4-dimensional system with beta initial guesses randomly distributed in the range [1, 12].

| run | $\alpha_{3}$ | $g_{31}$ | $g_{32}$ | $g_{33}$ | $g_{34}$ | $\beta_{3}$ | $h_{31}$ | $h_{32}$ | $h_{33}$ | $h_{34}$ | Error1 | Error2 |
| --- | --- | --- | --- | --- | --- | --- | --- | --- | --- | --- | --- | --- |
| 1 | 3.00 | 0.00 | 0.75 | 0.00 | 0.00 | 5.00 | 0.00 | 0.00 | 0.50 | 0.20 | 1.3382E-19 | 0.00019535 |
| 2 | 3.00 | 0.00 | 0.75 | 0.00 | 0.00 | 5.00 | 0.00 | 0.00 | 0.50 | 0.20 | 6.74895E-20 | 0.00027805 |
| 3 | 8.20 | 0.01 | 0.53 | 0.17 | 0.06 | 10.19 | 0.01 | 0.21 | 0.38 | 0.14 | 6.13065E-09 | 0.00018067 |
| 4 | 3.00 | 0.00 | 0.75 | 0.00 | 0.00 | 5.00 | 0.00 | 0.00 | 0.50 | 0.20 | 6.97704E-19 | 0.00036691 |
| 5 | 3.00 | 0.00 | 0.75 | 0.00 | 0.00 | 5.00 | 0.00 | 0.00 | 0.50 | 0.20 | 5.41785E-19 | 0.0001907 |
| 6 | 8.71 | 0.01 | 0.52 | 0.18 | 0.06 | 10.70 | 0.01 | 0.22 | 0.37 | 0.14 | 6.42557E-09 | 0.00037083 |
| 7 | 10.02 | 0.01 | 0.50 | 0.19 | 0.07 | 12.00 | 0.01 | 0.24 | 0.36 | 0.14 | 6.98231E-09 | 0.00034534 |
| 8 | 10.02 | 0.01 | 0.50 | 0.19 | 0.07 | 12.00 | 0.01 | 0.24 | 0.36 | 0.14 | 6.99209E-09 | 0.00019033 |
| 9 | 3.00 | 0.00 | 0.75 | 0.00 | 0.00 | 5.00 | 0.00 | 0.00 | 0.50 | 0.20 | 9.17453E-19 | 0.00025419 |
| 10 | 9.63 | 0.01 | 0.51 | 0.19 | 0.07 | 11.62 | 0.01 | 0.23 | 0.37 | 0.14 | 6.84281E-09 | 226.631556 |

Table 10 – Result of the 10 runs for the variable *X*_3_ of the 4-dimensional system with beta initial guesses randomly distributed in the range [1, 12].

| run | $\alpha_{4}$ | $g_{41}$ | $g_{42}$ | $g_{43}$ | $g_{44}$ | $\beta_{4}$ | $h_{41}$ | $h_{42}$ | $h_{43}$ | $h_{44}$ | Error1 | Error2 |
| --- | --- | --- | --- | --- | --- | --- | --- | --- | --- | --- | --- | --- |
| 1 | 11.73 | -0.96 | 2.00 | 2.03 | 1.04 | 12.00 | -0.99 | 2.00 | 2.00 | 1.06 | 0.0044895 | 3.016E-05 |
| 2 | 9.55 | -0.39 | 1.99 | 2.03 | 0.99 | 9.80 | -0.42 | 2.00 | 2.00 | 1.01 | 0.0021321 | 3.615E-05 |
| 3 | 10.09 | -0.94 | 2.00 | 2.03 | 1.04 | 10.36 | -0.97 | 2.00 | 2.00 | 1.05 | 0.0044025 | 2.98E-05 |
| 4 | 8.31 | -0.38 | 1.98 | 2.00 | 0.97 | 8.56 | -0.41 | 1.99 | 1.97 | 0.99 | 0.0024897 | 4.05E-05 |
| 5 | 9.20 | -0.97 | 2.00 | 2.03 | 1.04 | 9.47 | -1.00 | 2.00 | 2.00 | 1.06 | 0.004557 | 2.981E-05 |
| 6 | 11.75 | -0.40 | 2.00 | 2.03 | 0.99 | 12.00 | -0.43 | 2.00 | 2.00 | 1.00 | 0.0022829 | 4.058E-05 |
| 7 | 8.79 | -0.46 | 1.96 | 1.98 | 0.98 | 9.06 | -0.50 | 1.97 | 1.94 | 0.99 | 0.0024303 | 3.782E-05 |
| 8 | 11.73 | -0.98 | 2.00 | 2.03 | 1.04 | 12.00 | -1.00 | 2.00 | 2.00 | 1.06 | 0.0045649 | 2.98E-05 |
| 9 | 9.08 | -0.92 | 2.00 | 2.04 | 1.03 | 9.35 | -0.96 | 2.00 | 2.00 | 1.05 | 0.0043959 | 3.196E-05 |
| 10 | 11.75 | -0.45 | 2.00 | 2.03 | 0.99 | 12.00 | -0.48 | 2.00 | 2.00 | 1.00 | 0.002655 | 1.5577504 |

Table 11 – Result of the 10 runs for the variable *X*_4_ of the 4-dimensional system with beta initial guesses randomly distributed in the range [1, 12].

**Data set 3**

Tables 12-15 show the parameters found with the proposed algorithm for the 4-dimensional system (Equation 2) using the third set of initial values of the Table 1.

| run | $\alpha_{1}$ | $g_{11}$ | $g_{12}$ | $g_{13}$ | $g_{14}$ | $\beta_{1}$ | $h_{11}$ | $h_{12}$ | $h_{13}$ | $h_{14}$ | Error1 | Error2 |
| --- | --- | --- | --- | --- | --- | --- | --- | --- | --- | --- | --- | --- |
| 1 | 12.00 | 0.00 | 0.00 | -0.80 | 0.00 | 10.00 | 0.50 | 0.00 | 0.00 | 0.00 | 1.951E-19 | 5.823E-05 |
| 2 | 12.00 | 0.00 | 0.00 | -0.80 | 0.00 | 10.00 | 0.50 | 0.00 | 0.00 | 0.00 | 9.255E-16 | 4.841E-05 |
| 3 | 12.00 | 0.00 | 0.00 | -0.80 | 0.00 | 10.00 | 0.50 | 0.00 | 0.00 | 0.00 | 4.751E-19 | 3.1E-05 |
| 4 | 12.00 | 0.00 | 0.00 | -0.80 | 0.00 | 10.00 | 0.50 | 0.00 | 0.00 | 0.00 | 3.048E-19 | 4.958E-05 |
| 5 | 12.00 | 0.00 | 0.00 | -0.80 | 0.00 | 10.00 | 0.50 | 0.00 | 0.00 | 0.00 | 1.124E-18 | 4.356E-05 |
| 6 | 12.00 | 0.00 | 0.00 | -0.80 | 0.00 | 10.00 | 0.50 | 0.00 | 0.00 | 0.00 | 1.37E-17 | 4.339E-05 |
| 7 | 12.00 | 0.00 | 0.00 | -0.80 | 0.00 | 10.00 | 0.50 | 0.00 | 0.00 | 0.00 | 1.413E-18 | 3.998E-05 |
| 8 | 12.00 | 0.00 | 0.00 | -0.80 | 0.00 | 10.00 | 0.50 | 0.00 | 0.00 | 0.00 | 1.467E-18 | 5.818E-05 |
| 9 | 12.00 | 0.00 | 0.00 | -0.80 | 0.00 | 10.00 | 0.50 | 0.00 | 0.00 | 0.00 | 3.938E-20 | 5.089E-05 |
| 10 | 12.00 | 0.00 | 0.00 | -0.80 | 0.00 | 10.00 | 0.50 | 0.00 | 0.00 | 0.00 | 1.562E-18 | 4.242E-05 |

Table 12 – Result of the 10 runs for the variable *X*_1_ of the 4-dimensional system with beta initial guesses randomly distributed in the range [1, 12].

| run | $\alpha_{2}$ | $g_{21}$ | $g_{22}$ | $g_{23}$ | $g_{24}$ | $\beta_{2}$ | $h_{21}$ | $h_{22}$ | $h_{23}$ | $h_{24}$ | Error1 | Error2 |
| --- | --- | --- | --- | --- | --- | --- | --- | --- | --- | --- | --- | --- |
| 1 | 8.00 | 0.50 | 0.00 | 0.00 | 0.00 | 3.00 | 0.00 | 0.75 | 0.00 | 0.00 | 5.63E-21 | 8.603E-05 |
| 2 | 8.00 | 0.50 | 0.00 | 0.00 | 0.00 | 3.00 | 0.00 | 0.75 | 0.00 | 0.00 | 8.991E-20 | 8.813E-05 |
| 3 | 13.54 | 0.35 | 0.18 | 0.01 | 0.04 | 8.27 | 0.11 | 0.55 | 0.01 | 0.03 | 0.0015825 | 0.0001144 |
| 4 | 8.00 | 0.50 | 0.00 | 0.00 | 0.00 | 3.00 | 0.00 | 0.75 | 0.00 | 0.00 | 8.388E-19 | 8.719E-05 |
| 5 | 14.90 | 0.34 | 0.19 | 0.01 | 0.04 | 9.63 | 0.13 | 0.52 | 0.01 | 0.03 | 0.0015445 | 0.0001797 |
| 6 | 8.00 | 0.50 | 0.00 | 0.00 | 0.00 | 3.00 | 0.00 | 0.75 | 0.00 | 0.00 | 4.979E-18 | 5.506E-05 |
| 7 | 14.35 | 0.35 | 0.18 | 0.01 | 0.04 | 9.12 | 0.12 | 0.53 | 0.01 | 0.03 | 0.0015631 | 0.0001295 |
| 8 | 8.00 | 0.50 | 0.00 | 0.00 | 0.00 | 3.00 | 0.00 | 0.75 | 0.00 | 0.00 | 1.828E-20 | 8.692E-05 |
| 9 | 8.00 | 0.50 | 0.00 | 0.00 | 0.00 | 3.00 | 0.00 | 0.75 | 0.00 | 0.00 | 8.851E-19 | 8.772E-05 |
| 10 | 8.00 | 0.50 | 0.00 | 0.00 | 0.00 | 3.00 | 0.00 | 0.75 | 0.00 | 0.00 | 5.941E-19 | 5.469E-05 |

Table 13 – Result of the 10 runs for the variable *X*_2_ of the 4-dimensional system with beta initial guesses randomly distributed in the range [1, 12].

| run | $\alpha_{3}$ | $g_{31}$ | $g_{32}$ | $g_{33}$ | $g_{34}$ | $\beta_{3}$ | $h_{31}$ | $h_{32}$ | $h_{33}$ | $h_{34}$ | Error1 | Error2 |
| --- | --- | --- | --- | --- | --- | --- | --- | --- | --- | --- | --- | --- |
| 1 | 6.75 | 0.00 | 0.57 | 0.14 | 0.06 | 8.80 | 0.01 | 0.18 | 0.40 | 0.17 | 8.261E-06 | 0.0001034 |
| 2 | 5.96 | 0.00 | 0.59 | 0.12 | 0.05 | 8.00 | 0.00 | 0.16 | 0.41 | 0.17 | 7.036E-06 | 0.0001183 |
| 3 | 9.94 | 0.00 | 0.51 | 0.18 | 0.08 | 11.99 | 0.01 | 0.24 | 0.36 | 0.15 | 1.11E-05 | 6.29E-05 |
| 4 | 7.73 | 0.00 | 0.55 | 0.15 | 0.07 | 9.78 | 0.01 | 0.20 | 0.39 | 0.16 | 9.651E-06 | 8.188E-05 |
| 5 | 6.92 | 0.00 | 0.56 | 0.14 | 0.06 | 8.97 | 0.01 | 0.19 | 0.40 | 0.16 | 8.602E-06 | 0.0001291 |
| 6 | 9.95 | 0.00 | 0.51 | 0.18 | 0.08 | 12.00 | 0.01 | 0.24 | 0.36 | 0.15 | 1.153E-05 | 8.57E-05 |
| 7 | 8.91 | 0.00 | 0.53 | 0.17 | 0.07 | 10.97 | 0.00 | 0.23 | 0.37 | 0.16 | 1.009E-05 | 9.786E-05 |
| 8 | 9.94 | 0.00 | 0.51 | 0.18 | 0.08 | 12.00 | 0.01 | 0.24 | 0.36 | 0.15 | 1.119E-05 | 0.0001227 |
| 9 | 7.04 | 0.00 | 0.56 | 0.14 | 0.06 | 9.08 | 0.01 | 0.19 | 0.39 | 0.16 | 8.764E-06 | 9.221E-05 |
| 10 | 9.95 | 0.00 | 0.51 | 0.18 | 0.08 | 12.00 | 0.01 | 0.24 | 0.36 | 0.15 | 1.154E-05 | 0.0001006 |

Table 14 – Result of the 10 runs for the variable *X*_3_ of the 4-dimensional system with beta initial guesses randomly distributed in the range [1, 12].

| run | $\alpha_{4}$ | $g_{41}$ | $g_{42}$ | $g_{43}$ | $g_{44}$ | $\beta_{4}$ | $h_{41}$ | $h_{42}$ | $h_{43}$ | $h_{44}$ | Error1 | Error2 |
| --- | --- | --- | --- | --- | --- | --- | --- | --- | --- | --- | --- | --- |
| 1 | 8.00 | 0.34 | -0.03 | 0.02 | 0.26 | 12.00 | 0.16 | -0.03 | 0.02 | 0.56 | 2.541E-07 | 1.381E-06 |
| 2 | 8.00 | 0.34 | -0.02 | 0.02 | 0.27 | 12.00 | 0.16 | -0.03 | 0.02 | 0.56 | 3.332E-07 | 1.369E-06 |
| 3 | 2.00 | 0.50 | 0.00 | 0.00 | 0.00 | 6.00 | 0.00 | 0.00 | 0.00 | 0.80 | 7.707E-20 | 1.272E-06 |
| 4 | 8.00 | 0.34 | -0.02 | 0.02 | 0.27 | 12.00 | 0.16 | -0.03 | 0.02 | 0.56 | 3.232E-07 | 1.214E-06 |
| 5 | 8.00 | 0.34 | -0.02 | 0.02 | 0.27 | 12.00 | 0.16 | -0.03 | 0.02 | 0.56 | 3.139E-07 | 1.327E-06 |
| 6 | 8.00 | 0.34 | -0.02 | 0.02 | 0.27 | 12.00 | 0.16 | -0.03 | 0.02 | 0.56 | 3.147E-07 | 1.291E-06 |
| 7 | 8.00 | 0.34 | -0.02 | 0.02 | 0.27 | 12.00 | 0.16 | -0.03 | 0.02 | 0.56 | 3.352E-07 | 1.283E-06 |
| 8 | 8.00 | 0.34 | -0.02 | 0.02 | 0.27 | 12.00 | 0.16 | -0.03 | 0.02 | 0.56 | 3.315E-07 | 1.503E-06 |
| 9 | 8.00 | 0.34 | -0.02 | 0.02 | 0.27 | 12.00 | 0.16 | -0.03 | 0.02 | 0.56 | 3.14E-07 | 1.245E-06 |
| 10 | 2.00 | 0.50 | 0.00 | 0.00 | 0.00 | 6.00 | 0.00 | 0.00 | 0.00 | 0.80 | 8.869E-23 | 1.049E-06 |

Table 15 – Result of the 10 runs for the variable *X*_4_ of the 4-dimensional system with beta initial guesses randomly distributed in the range [1, 12].

## 5-Dimensional system results – noise-free time series

**Data set 1**

Tables 16-20 show the parameters found with the proposed algorithm for the 5-dimensional system (Equation 3) using the first set of initial values of the Table 2. The time series used in this case study were obtained by numerical integration of the 5-dimensional system in the interval [0,5] with 0.1 sampling interval.

| run | $\alpha_{1}$ | $g_{11}$ | $g_{12}$ | $g_{13}$ | $g_{14}$ | $g_{15}$ | $\beta_{1}$ | $h_{11}$ | $h_{12}$ | $h_{13}$ | $h_{14}$ | $h_{15}$ | Error1 | Error2 |
| --- | --- | --- | --- | --- | --- | --- | --- | --- | --- | --- | --- | --- | --- | --- |
| 1 | 5.00 | 0.00 | 0.00 | 1.00 | 0.00 | -1.00 | 10.00 | 2.00 | 0.00 | 0.00 | 0.00 | 0.00 | 1.22343E-21 | 7.99402E-21 |
| 2 | 5.00 | 0.00 | 0.00 | 1.00 | 0.00 | -1.00 | 10.00 | 2.00 | 0.00 | 0.00 | 0.00 | 0.00 | 1.32077E-21 | 4.59591E-21 |
| 3 | 5.00 | 0.00 | 0.00 | 1.00 | 0.00 | -1.00 | 10.00 | 2.00 | 0.00 | 0.00 | 0.00 | 0.00 | 1.32077E-21 | 3.6282E-21 |
| 4 | 5.00 | 0.00 | 0.00 | 1.00 | 0.00 | -1.00 | 10.00 | 2.00 | 0.00 | 0.00 | 0.00 | 0.00 | 1.32077E-21 | 4.03837E-21 |
| 5 | 5.00 | 0.00 | 0.00 | 1.00 | 0.00 | -1.00 | 10.00 | 2.00 | 0.00 | 0.00 | 0.00 | 0.00 | 1.32077E-21 | 2.72773E-21 |
| 6 | 5.00 | 0.00 | 0.00 | 1.00 | 0.00 | -1.00 | 10.00 | 2.00 | 0.00 | 0.00 | 0.00 | 0.00 | 5.15785E-22 | 4.19796E-21 |
| 7 | 5.00 | 0.00 | 0.00 | 1.00 | 0.00 | -1.00 | 10.00 | 2.00 | 0.00 | 0.00 | 0.00 | 0.00 | 1.05374E-20 | 5.19914E-21 |
| 8 | 5.00 | 0.00 | 0.00 | 1.00 | 0.00 | -1.00 | 10.00 | 2.00 | 0.00 | 0.00 | 0.00 | 0.00 | 2.22619E-21 | 1.58118E-20 |
| 9 | 5.00 | 0.00 | 0.00 | 1.00 | 0.00 | -1.00 | 10.00 | 2.00 | 0.00 | 0.00 | 0.00 | 0.00 | 6.3629E-22 | 3.98716E-07 |
| 10 | 5.00 | 0.00 | 0.00 | 1.00 | 0.00 | -1.00 | 10.00 | 2.00 | 0.00 | 0.00 | 0.00 | 0.00 | 1.83537E-21 | 5.99782E-21 |

Table 16 – Result of the 10 runs for the variable *X*_1_ of the 5-dimensional system with beta initial guesses uniformly distributed in the range [1, 10].

| run | $\alpha_{2}$ | $g_{21}$ | $g_{22}$ | $g_{23}$ | $g_{24}$ | $g_{25}$ | $\beta_{2}$ | $h_{21}$ | $h_{22}$ | $h_{23}$ | $h_{24}$ | $h_{25}$ | Error1 | Error2 |
| --- | --- | --- | --- | --- | --- | --- | --- | --- | --- | --- | --- | --- | --- | --- |
| 1 | 10.00 | 2.00 | 0.00 | 0.00 | 0.00 | 0.00 | 10.00 | 0.00 | 2.00 | 0.00 | 0.00 | 0.00 | 1.58004E-18 | 1.26958E-20 |
| 2 | 10.00 | 2.00 | 0.00 | 0.00 | 0.00 | 0.00 | 10.00 | 0.00 | 2.00 | 0.00 | 0.00 | 0.00 | 6.21989E-19 | 5.93212E-21 |
| 3 | 10.00 | 2.00 | 0.00 | 0.00 | 0.00 | 0.00 | 10.00 | 0.00 | 2.00 | 0.00 | 0.00 | 0.00 | 2.40218E-18 | 1.14226E-20 |
| 4 | 10.00 | 2.00 | 0.00 | 0.00 | 0.00 | 0.00 | 10.00 | 0.00 | 2.00 | 0.00 | 0.00 | 0.00 | 3.48423E-18 | 1.03853E-20 |
| 5 | 10.00 | 2.00 | 0.00 | 0.00 | 0.00 | 0.00 | 10.00 | 0.00 | 2.00 | 0.00 | 0.00 | 0.00 | 1.31001E-18 | 5.43993E-21 |
| 6 | 10.00 | 2.00 | 0.00 | 0.00 | 0.00 | 0.00 | 10.00 | 0.00 | 2.00 | 0.00 | 0.00 | 0.00 | 1.31001E-18 | 9.8609E-21 |
| 7 | 10.00 | 2.00 | 0.00 | 0.00 | 0.00 | 0.00 | 10.00 | 0.00 | 2.00 | 0.00 | 0.00 | 0.00 | 1.31001E-18 | 7.29945E-21 |
| 8 | 10.00 | 2.00 | 0.00 | 0.00 | 0.00 | 0.00 | 10.00 | 0.00 | 2.00 | 0.00 | 0.00 | 0.00 | 6.02476E-19 | 8.94865E-21 |
| 9 | 10.00 | 2.00 | 0.00 | 0.00 | 0.00 | 0.00 | 10.00 | 0.00 | 2.00 | 0.00 | 0.00 | 0.00 | 3.64807E-18 | 1.20049E-07 |
| 10 | 10.00 | 2.00 | 0.00 | 0.00 | 0.00 | 0.00 | 10.00 | 0.00 | 2.00 | 0.00 | 0.00 | 0.00 | 4.91918E-18 | 3.01878E-20 |

Table 17 – Result of the 10 runs for the variable *X*_2_ of the 5-dimensional system with beta initial guesses uniformly distributed in the range [1, 10].

| run | $\alpha_{3}$ | $g_{31}$ | $g_{32}$ | $g_{33}$ | $g_{34}$ | $g_{35}$ | $\beta_{3}$ | $h_{31}$ | $h_{32}$ | $h_{33}$ | $h_{34}$ | $h_{35}$ | Error1 | Error2 |
| --- | --- | --- | --- | --- | --- | --- | --- | --- | --- | --- | --- | --- | --- | --- |
| 1 | 10.00 | 0.00 | -1.00 | 0.00 | 0.00 | 0.00 | 10.00 | 0.00 | -1.00 | 2.00 | 0.00 | 0.00 | 1.28105E-20 | 2.16667E-22 |
| 2 | 10.00 | 0.00 | -1.00 | 0.00 | 0.00 | 0.00 | 10.00 | 0.00 | -1.00 | 2.00 | 0.00 | 0.00 | 1.28105E-20 | 7.2618E-23 |
| 3 | 10.00 | 0.00 | -1.00 | 0.00 | 0.00 | 0.00 | 10.00 | 0.00 | -1.00 | 2.00 | 0.00 | 0.00 | 1.28105E-20 | 2.00922E-22 |
| 4 | 10.00 | 0.00 | -1.00 | 0.00 | 0.00 | 0.00 | 10.00 | 0.00 | -1.00 | 2.00 | 0.00 | 0.00 | 1.28105E-20 | 2.0588E-22 |
| 5 | 10.00 | 0.00 | -1.00 | 0.00 | 0.00 | 0.00 | 10.00 | 0.00 | -1.00 | 2.00 | 0.00 | 0.00 | 1.28105E-20 | 1.62388E-22 |
| 6 | 10.00 | 0.00 | -1.00 | 0.00 | 0.00 | 0.00 | 10.00 | 0.00 | -1.00 | 2.00 | 0.00 | 0.00 | 1.28105E-20 | 1.64324E-22 |
| 7 | 10.00 | 0.00 | -1.00 | 0.00 | 0.00 | 0.00 | 10.00 | 0.00 | -1.00 | 2.00 | 0.00 | 0.00 | 1.03057E-19 | 1.04365E-22 |
| 8 | 10.00 | 0.00 | -1.00 | 0.00 | 0.00 | 0.00 | 10.00 | 0.00 | -1.00 | 2.00 | 0.00 | 0.00 | 3.29855E-21 | 3.31941E-22 |
| 9 | 10.00 | 0.00 | -1.00 | 0.00 | 0.00 | 0.00 | 10.00 | 0.00 | -1.00 | 2.00 | 0.00 | 0.00 | 1.82851E-21 | 1.44575E-09 |
| 10 | 10.00 | 0.00 | -1.00 | 0.00 | 0.00 | 0.00 | 10.00 | 0.00 | -1.00 | 2.00 | 0.00 | 0.00 | 2.01525E-19 | 2.13752E-21 |

Table 18 – Result of the 10 runs for the variable *X*_3_ of the 5-dimensional system with beta initial guesses uniformly distributed in the range [1, 10].

| Run | $\alpha_{4}$ | $g_{41}$ | $g_{42}$ | $g_{43}$ | $g_{44}$ | $g_{45}$ | $\beta_{4}$ | $h_{41}$ | $h_{42}$ | $h_{43}$ | $h_{44}$ | $h_{45}$ | Error1 | Error2 |
| --- | --- | --- | --- | --- | --- | --- | --- | --- | --- | --- | --- | --- | --- | --- |
| 1 | 8.00 | 0.00 | 0.00 | 2.00 | 0.00 | -1.00 | 10.00 | 0.00 | 0.00 | 0.00 | 2.00 | 0.00 | 2.9075E-21 | 2.58337E-21 |
| 2 | 8.00 | 0.00 | 0.00 | 2.00 | 0.00 | -1.00 | 10.00 | 0.00 | 0.00 | 0.00 | 2.00 | 0.00 | 8.9608E-22 | 4.95432E-21 |
| 3 | 8.00 | 0.00 | 0.00 | 2.00 | 0.00 | -1.00 | 10.00 | 0.00 | 0.00 | 0.00 | 2.00 | 0.00 | 8.1267E-22 | 4.23046E-21 |
| 4 | 8.00 | 0.00 | 0.00 | 2.00 | 0.00 | -1.00 | 10.00 | 0.00 | 0.00 | 0.00 | 2.00 | 0.00 | 8.1267E-22 | 4.83447E-21 |
| 5 | 8.00 | 0.00 | 0.00 | 2.00 | 0.00 | -1.00 | 10.00 | 0.00 | 0.00 | 0.00 | 2.00 | 0.00 | 8.1267E-22 | 5.07495E-21 |
| 6 | 8.00 | 0.00 | 0.00 | 2.00 | 0.00 | -1.00 | 10.00 | 0.00 | 0.00 | 0.00 | 2.00 | 0.00 | 8.1267E-22 | 6.43373E-21 |
| 7 | 8.00 | 0.00 | 0.00 | 2.00 | 0.00 | -1.00 | 10.00 | 0.00 | 0.00 | 0.00 | 2.00 | 0.00 | 7.7966E-22 | 2.41294E-21 |
| 8 | 8.00 | 0.00 | 0.00 | 2.00 | 0.00 | -1.00 | 10.00 | 0.00 | 0.00 | 0.00 | 2.00 | 0.00 | 5.8343E-20 | 2.24091E-20 |
| 9 | 8.00 | 0.00 | 0.00 | 2.00 | 0.00 | -1.00 | 10.00 | 0.00 | 0.00 | 0.00 | 2.00 | 0.00 | 1.5997E-21 | 6.49168E-07 |
| 10 | 8.00 | 0.00 | 0.00 | 2.00 | 0.00 | -1.00 | 10.00 | 0.00 | 0.00 | 0.00 | 2.00 | 0.00 | 3.5062E-21 | 8.42201E-21 |

Table 19 – Result of the 10 runs for the variable *X*_4_ of the 5-dimensional system with beta initial guesses uniformly distributed in the range [1, 10].

| run | $\alpha_{5}$ | $g_{51}$ | $g_{52}$ | $g_{53}$ | $g_{54}$ | $g_{55}$ | $\beta_{5}$ | $h_{51}$ | $h_{52}$ | $h_{53}$ | $h_{54}$ | $h_{55}$ | Error1 | Error2 |
| --- | --- | --- | --- | --- | --- | --- | --- | --- | --- | --- | --- | --- | --- | --- |
| 1 | 10.00 | 0.00 | 0.00 | 0.00 | 2.00 | 0.00 | 10.00 | 0.00 | 0.00 | 0.00 | 0.00 | 2.00 | 1.0044E-18 | 1.64228E-21 |
| 2 | 10.00 | 0.00 | 0.00 | 0.00 | 2.00 | 0.00 | 10.00 | 0.00 | 0.00 | 0.00 | 0.00 | 2.00 | 1.0044E-18 | 2.32214E-21 |
| 3 | 10.00 | 0.00 | 0.00 | 0.00 | 2.00 | 0.00 | 10.00 | 0.00 | 0.00 | 0.00 | 0.00 | 2.00 | 1.0044E-18 | 2.16712E-21 |
| 4 | 10.00 | 0.00 | 0.00 | 0.00 | 2.00 | 0.00 | 10.00 | 0.00 | 0.00 | 0.00 | 0.00 | 2.00 | 1.0044E-18 | 2.7372E-21 |
| 5 | 10.00 | 0.00 | 0.00 | 0.00 | 2.00 | 0.00 | 10.00 | 0.00 | 0.00 | 0.00 | 0.00 | 2.00 | 5.1333E-19 | 2.84682E-21 |
| 6 | 10.00 | 0.00 | 0.00 | 0.00 | 2.00 | 0.00 | 10.00 | 0.00 | 0.00 | 0.00 | 0.00 | 2.00 | 6.2861E-19 | 2.81858E-21 |
| 7 | 10.00 | 0.00 | 0.00 | 0.00 | 2.00 | 0.00 | 10.00 | 0.00 | 0.00 | 0.00 | 0.00 | 2.00 | 3.4288E-19 | 1.1659E-21 |
| 8 | 10.00 | 0.00 | 0.00 | 0.00 | 2.00 | 0.00 | 10.00 | 0.00 | 0.00 | 0.00 | 0.00 | 2.00 | 3.9076E-20 | 1.14064E-20 |
| 9 | 9.15 | -0.60 | 0.07 | -0.29 | 2.59 | 0.04 | 10.96 | 0.56 | -0.23 | -1.00 | -0.51 | 2.00 | 0.00011205 | 3.68627E-07 |
| 10 | 10.00 | 0.00 | 0.00 | 0.00 | 2.00 | 0.00 | 10.00 | 0.00 | 0.00 | 0.00 | 0.00 | 2.00 | 6.7383E-23 | 3.01361E-21 |

Table 20 – Result of the 10 runs for the variable *X*_5_ of the 5-dimensional system with beta initial guesses uniformly distributed in the range [1, 10].

**Data set 2**

Tables 21-25 show the parameters found with the proposed algorithm for the 5-dimensional system (Equation 3) using the second set of initial values of the Table 2.

| run | $\alpha_{1}$ | $g_{11}$ | $g_{12}$ | $g_{13}$ | $g_{14}$ | $g_{15}$ | $\beta_{1}$ | $h_{11}$ | $h_{12}$ | $h_{13}$ | $h_{14}$ | $h_{15}$ | Error1 | Error2 |
| --- | --- | --- | --- | --- | --- | --- | --- | --- | --- | --- | --- | --- | --- | --- |
| 1 | 5.00 | 0.00 | 0.00 | 1.00 | 0.00 | -1.00 | 10.00 | 2.00 | 0.00 | 0.00 | 0.00 | 0.00 | 1.1897E-21 | 1.01859158 |
| 2 | 5.00 | 0.00 | 0.00 | 1.00 | 0.00 | -1.00 | 10.00 | 2.00 | 0.00 | 0.00 | 0.00 | 0.00 | 1.1897E-21 | 1.01859158 |
| 3 | 5.00 | 0.00 | 0.00 | 1.00 | 0.00 | -1.00 | 10.00 | 2.00 | 0.00 | 0.00 | 0.00 | 0.00 | 1.1897E-21 | 1.01859158 |
| 4 | 5.00 | 0.00 | 0.00 | 1.00 | 0.00 | -1.00 | 10.00 | 2.00 | 0.00 | 0.00 | 0.00 | 0.00 | 2.5682E-19 | 1.01859158 |
| 5 | 5.00 | 0.00 | 0.00 | 1.00 | 0.00 | -1.00 | 10.00 | 2.00 | 0.00 | 0.00 | 0.00 | 0.00 | 2.9345E-19 | 1.01859158 |
| 6 | 5.00 | 0.00 | 0.00 | 1.00 | 0.00 | -1.00 | 10.00 | 2.00 | 0.00 | 0.00 | 0.00 | 0.00 | 1.016E-19 | 1.01859158 |
| 7 | 5.00 | 0.00 | 0.00 | 1.00 | 0.00 | -1.00 | 10.00 | 2.00 | 0.00 | 0.00 | 0.00 | 0.00 | 1.8676E-21 | 1.01920181 |
| 8 | 5.00 | 0.00 | 0.00 | 1.00 | 0.00 | -1.00 | 10.00 | 2.00 | 0.00 | 0.00 | 0.00 | 0.00 | 3.7676E-21 | 1.01851419 |
| 9 | 5.00 | 0.00 | 0.00 | 1.00 | 0.00 | -1.00 | 10.00 | 2.00 | 0.00 | 0.00 | 0.00 | 0.00 | 1.2111E-19 | 1.02365495 |
| 10 | 5.00 | 0.00 | 0.00 | 1.00 | 0.00 | -1.00 | 10.00 | 2.00 | 0.00 | 0.00 | 0.00 | 0.00 | 3.5407E-21 | 1.01721472 |

Table 21 – Result of the 10 runs for the variable *X*_1_ of the 5-dimensional system with beta initial guesses uniformly distributed in the range [1, 10].

| run | $\alpha_{2}$ | $g_{21}$ | $g_{22}$ | $g_{23}$ | $g_{24}$ | $g_{25}$ | $\beta_{2}$ | $h_{21}$ | $h_{22}$ | $h_{23}$ | $h_{24}$ | $h_{25}$ | Error1 | Error2 |
| --- | --- | --- | --- | --- | --- | --- | --- | --- | --- | --- | --- | --- | --- | --- |
| 1 | 10.00 | 2.00 | 0.00 | 0.00 | 0.00 | 0.00 | 10.00 | 0.00 | 2.00 | 0.00 | 0.00 | 0.00 | 4.49185E-19 | 1.19113792 |
| 2 | 10.00 | 2.00 | 0.00 | 0.00 | 0.00 | 0.00 | 10.00 | 0.00 | 2.00 | 0.00 | 0.00 | 0.00 | 4.49185E-19 | 1.19113792 |
| 3 | 10.00 | 2.00 | 0.00 | 0.00 | 0.00 | 0.00 | 10.00 | 0.00 | 2.00 | 0.00 | 0.00 | 0.00 | 4.49185E-19 | 1.19113792 |
| 4 | 10.00 | 2.00 | 0.00 | 0.00 | 0.00 | 0.00 | 10.00 | 0.00 | 2.00 | 0.00 | 0.00 | 0.00 | 4.49185E-19 | 1.19113792 |
| 5 | 10.00 | 2.00 | 0.00 | 0.00 | 0.00 | 0.00 | 10.00 | 0.00 | 2.00 | 0.00 | 0.00 | 0.00 | 4.49185E-19 | 1.19113792 |
| 6 | 10.00 | 2.00 | 0.00 | 0.00 | 0.00 | 0.00 | 10.00 | 0.00 | 2.00 | 0.00 | 0.00 | 0.00 | 1.80194E-21 | 1.19113792 |
| 7 | 10.00 | 2.00 | 0.00 | 0.00 | 0.00 | 0.00 | 10.00 | 0.00 | 2.00 | 0.00 | 0.00 | 0.00 | 4.52709E-20 | 1.19101899 |
| 8 | 10.00 | 2.00 | 0.00 | 0.00 | 0.00 | 0.00 | 10.00 | 0.00 | 2.00 | 0.00 | 0.00 | 0.00 | 1.53429E-22 | 1.19101292 |
| 9 | 10.00 | 2.00 | 0.00 | 0.00 | 0.00 | 0.00 | 10.00 | 0.00 | 2.00 | 0.00 | 0.00 | 0.00 | 3.05238E-20 | 1.19595194 |
| 10 | 10.00 | 2.00 | 0.00 | 0.00 | 0.00 | 0.00 | 10.00 | 0.00 | 2.00 | 0.00 | 0.00 | 0.00 | 1.79649E-20 | 1.19060203 |

Table 22 – Result of the 10 runs for the variable *X*_2_ of the 5-dimensional system with beta initial guesses uniformly distributed in the range [1, 10].

| run | $\alpha_{3}$ | $g_{31}$ | $g_{32}$ | $g_{33}$ | $g_{34}$ | $g_{35}$ | $\beta_{3}$ | $h_{31}$ | $h_{32}$ | $h_{33}$ | $h_{34}$ | $h_{35}$ | Error1 | Error2 |
| --- | --- | --- | --- | --- | --- | --- | --- | --- | --- | --- | --- | --- | --- | --- |
| 1 | 5.98 | -0.79 | -1.06 | -0.03 | -0.38 | -0.10 | 5.79 | -1.00 | -1.00 | 0.02 | -0.17 | -0.09 | 3.029E-05 | 0.121 |
| 2 | 5.98 | -0.79 | -1.06 | -0.03 | -0.38 | -0.10 | 5.79 | -1.00 | -1.00 | 0.02 | -0.17 | -0.09 | 3.029E-05 | 0.121 |
| 3 | 5.98 | -0.79 | -1.06 | -0.03 | -0.38 | -0.10 | 5.79 | -1.00 | -1.00 | 0.02 | -0.17 | -0.09 | 3.029E-05 | 0.121 |
| 4 | 5.98 | -0.79 | -1.06 | -0.03 | -0.38 | -0.10 | 5.79 | -1.00 | -1.00 | 0.02 | -0.17 | -0.09 | 3.029E-05 | 0.121 |
| 5 | 5.98 | -0.79 | -1.06 | -0.03 | -0.38 | -0.10 | 5.79 | -1.00 | -1.00 | 0.02 | -0.17 | -0.09 | 3.029E-05 | 0.121 |
| 6 | 5.98 | -0.79 | -1.06 | -0.03 | -0.38 | -0.10 | 5.79 | -1.00 | -1.00 | 0.02 | -0.17 | -0.09 | 3.029E-05 | 0.121 |
| 7 | 12.10 | -0.94 | -1.02 | 0.16 | 0.08 | 0.00 | 12.00 | -1.00 | -1.00 | 1.17 | 0.15 | 0.00 | 1.8E-05 | 0.1223211 |
| 8 | 8.45 | -0.79 | -1.04 | -0.03 | -0.36 | -0.12 | 8.27 | -0.93 | -1.00 | 0.00 | -0.23 | -0.11 | 2.748E-05 | 0.1210038 |
| 9 | 9.12 | -0.98 | -0.99 | -0.37 | -0.99 | -0.36 | 9.07 | -1.00 | -1.00 | -0.38 | -1.00 | -0.30 | 0.006737 | 0.1251891 |
| 10 | 10.16 | -0.52 | -0.50 | -0.10 | -0.58 | -0.37 | 9.91 | -0.70 | -0.44 | -0.07 | -0.41 | -0.39 | 0.000513 | 0.1231134 |

Table 23 – Result of the 10 runs for the variable *X*_3_ of the 5-dimensional system with beta initial guesses uniformly distributed in the range [1, 10].

| run | $\alpha_{4}$ | $g_{41}$ | $g_{42}$ | $g_{43}$ | $g_{44}$ | $g_{45}$ | $\beta_{4}$ | $h_{41}$ | $h_{42}$ | $h_{43}$ | $h_{44}$ | $h_{45}$ | Error1 | Error2 |
| --- | --- | --- | --- | --- | --- | --- | --- | --- | --- | --- | --- | --- | --- | --- |
| 1 | 8.00 | 0.00 | 0.00 | 2.00 | 0.00 | -1.00 | 10.00 | 0.00 | 0.00 | 0.00 | 2.00 | 0.00 | 1.51027E-21 | 0.92879101 |
| 2 | 8.00 | 0.00 | 0.00 | 2.00 | 0.00 | -1.00 | 10.00 | 0.00 | 0.00 | 0.00 | 2.00 | 0.00 | 1.51027E-21 | 0.92879101 |
| 3 | 8.00 | 0.00 | 0.00 | 2.00 | 0.00 | -1.00 | 10.00 | 0.00 | 0.00 | 0.00 | 2.00 | 0.00 | 1.59811E-18 | 0.92879101 |
| 4 | 8.00 | 0.00 | 0.00 | 2.00 | 0.00 | -1.00 | 10.00 | 0.00 | 0.00 | 0.00 | 2.00 | 0.00 | 1.59811E-18 | 0.92879101 |
| 5 | 8.00 | 0.00 | 0.00 | 2.00 | 0.00 | -1.00 | 10.00 | 0.00 | 0.00 | 0.00 | 2.00 | 0.00 | 1.59811E-18 | 0.92879101 |
| 6 | 8.00 | 0.00 | 0.00 | 2.00 | 0.00 | -1.00 | 10.00 | 0.00 | 0.00 | 0.00 | 2.00 | 0.00 | 9.37109E-22 | 0.92879101 |
| 7 | 8.00 | 0.00 | 0.00 | 2.00 | 0.00 | -1.00 | 10.00 | 0.00 | 0.00 | 0.00 | 2.00 | 0.00 | 9.19898E-22 | 0.9325269 |
| 8 | 8.00 | 0.00 | 0.00 | 2.00 | 0.00 | -1.00 | 10.00 | 0.00 | 0.00 | 0.00 | 2.00 | 0.00 | 9.95E-22 | 0.92852558 |
| 9 | 8.00 | 0.00 | 0.00 | 2.00 | 0.00 | -1.00 | 10.00 | 0.00 | 0.00 | 0.00 | 2.00 | 0.00 | 4.29953E-21 | 0.94487667 |
| 10 | 8.00 | 0.00 | 0.00 | 2.00 | 0.00 | -1.00 | 10.00 | 0.00 | 0.00 | 0.00 | 2.00 | 0.00 | 3.74754E-21 | 0.92760596 |

Table 24 – Result of the 10 runs for the variable *X*_4_ of the 5-dimensional system with beta initial guesses uniformly distributed in the range [1, 10].

| run | $\alpha_{5}$ | $g_{51}$ | $g_{52}$ | $g_{53}$ | $g_{54}$ | $g_{55}$ | $\beta_{5}$ | $h_{51}$ | $h_{52}$ | $h_{53}$ | $h_{54}$ | $h_{55}$ | Error1 | Error2 |
| --- | --- | --- | --- | --- | --- | --- | --- | --- | --- | --- | --- | --- | --- | --- |
| 1 | 10.00 | 0.00 | 0.00 | 0.00 | 2.00 | 0.00 | 10.00 | 0.00 | 0.00 | 0.00 | 0.00 | 2.00 | 2.28777E-20 | 2.67857553 |
| 2 | 10.00 | 0.00 | 0.00 | 0.00 | 2.00 | 0.00 | 10.00 | 0.00 | 0.00 | 0.00 | 0.00 | 2.00 | 2.28777E-20 | 2.67857553 |
| 3 | 10.00 | 0.00 | 0.00 | 0.00 | 2.00 | 0.00 | 10.00 | 0.00 | 0.00 | 0.00 | 0.00 | 2.00 | 2.28777E-20 | 2.67857553 |
| 4 | 10.00 | 0.00 | 0.00 | 0.00 | 2.00 | 0.00 | 10.00 | 0.00 | 0.00 | 0.00 | 0.00 | 2.00 | 2.28777E-20 | 2.67857553 |
| 5 | 10.00 | 0.00 | 0.00 | 0.00 | 2.00 | 0.00 | 10.00 | 0.00 | 0.00 | 0.00 | 0.00 | 2.00 | 2.28777E-20 | 2.67857553 |
| 6 | 10.00 | 0.00 | 0.00 | 0.00 | 2.00 | 0.00 | 10.00 | 0.00 | 0.00 | 0.00 | 0.00 | 2.00 | 2.28777E-20 | 2.67857553 |
| 7 | 10.00 | 0.00 | 0.00 | 0.00 | 2.00 | 0.00 | 10.00 | 0.00 | 0.00 | 0.00 | 0.00 | 2.00 | 3.34897E-18 | 2.68183717 |
| 8 | 10.00 | 0.00 | 0.00 | 0.00 | 2.00 | 0.00 | 10.00 | 0.00 | 0.00 | 0.00 | 0.00 | 2.00 | 3.28198E-18 | 2.6784461 |
| 9 | 10.00 | 0.00 | 0.00 | 0.00 | 2.00 | 0.00 | 10.00 | 0.00 | 0.00 | 0.00 | 0.00 | 2.00 | 1.50711E-18 | 2.67915613 |
| 10 | 10.00 | 0.00 | 0.00 | 0.00 | 2.00 | 0.00 | 10.00 | 0.00 | 0.00 | 0.00 | 0.00 | 2.00 | 1.37099E-18 | 2.68537022 |

Table 25 – Result of the 10 runs for the variable *X*_5_ of the 5-dimensional system with beta initial guesses uniformly distributed in the range [1, 10].

**Data set 3**

Tables 26-30 show the parameters found with the proposed algorithm for the 5-dimensional system (Equation 3) using the third set of initial values of the Table 2.

| run | $\alpha_{1}$ | $g_{11}$ | $g_{12}$ | $g_{13}$ | $g_{14}$ | $g_{15}$ | $\beta_{1}$ | $h_{11}$ | $h_{12}$ | $h_{13}$ | $h_{14}$ | $h_{15}$ | Error1 | Error2 |
| --- | --- | --- | --- | --- | --- | --- | --- | --- | --- | --- | --- | --- | --- | --- |
| 1 | 4.99 | -0.80 | 1.90 | -0.50 | 0.13 | -1.70 | 12.00 | 1.91 | 1.90 | -0.28 | -0.54 | -0.47 | 3.65603E-05 | 9.0543E-06 |
| 2 | 5.01 | -0.77 | 1.85 | -0.50 | 0.12 | -1.68 | 12.00 | 1.92 | 1.85 | -0.27 | -0.54 | -0.46 | 3.65176E-05 | 1.3913E-05 |
| 3 | 4.98 | -0.79 | 1.87 | -0.50 | 0.13 | -1.69 | 11.97 | 1.92 | 1.86 | -0.28 | -0.54 | -0.46 | 3.67547E-05 | 1.1783E-05 |
| 4 | 5.02 | -0.79 | 1.87 | -0.50 | 0.13 | -1.68 | 12.00 | 1.91 | 1.86 | -0.28 | -0.54 | -0.47 | 3.68794E-05 | 1.1749E-05 |
| 5 | 5.02 | -0.79 | 1.87 | -0.50 | 0.13 | -1.68 | 12.00 | 1.91 | 1.86 | -0.28 | -0.54 | -0.47 | 3.68794E-05 | 1.1598E-05 |
| 6 | 5.02 | -0.79 | 1.87 | -0.50 | 0.13 | -1.68 | 12.00 | 1.91 | 1.86 | -0.28 | -0.54 | -0.47 | 3.68794E-05 | 1.1598E-05 |
| 7 | 5.02 | -0.78 | 1.87 | -0.50 | 0.13 | -1.68 | 12.00 | 1.91 | 1.86 | -0.28 | -0.54 | -0.47 | 3.67527E-05 | 1.1808E-05 |
| 8 | 5.00 | 0.00 | 0.00 | 1.00 | 0.00 | -1.00 | 10.00 | 2.00 | 0.00 | 0.00 | 0.00 | 0.00 | 1.29656E-20 | 0.00283704 |
| 9 | 5.00 | 0.00 | 0.00 | 1.00 | 0.00 | -1.00 | 10.00 | 2.00 | 0.00 | 0.00 | 0.00 | 0.00 | 1.24481E-21 | 8.8742E-06 |
| 10 | 5.00 | 0.00 | 0.00 | 1.00 | 0.00 | -1.00 | 10.00 | 2.00 | 0.00 | 0.00 | 0.00 | 0.00 | 1.29811E-20 | 1.9804E-05 |

Table 26 – Result of the 10 runs for the variable *X*_1_ of the 5-dimensional system with beta initial guesses uniformly distributed in the range [1, 10].

| run | $\alpha_{2}$ | $g_{21}$ | $g_{22}$ | $g_{23}$ | $g_{24}$ | $g_{25}$ | $\beta_{2}$ | $h_{21}$ | $h_{22}$ | $h_{23}$ | $h_{24}$ | $h_{25}$ | Error1 | Error2 |
| --- | --- | --- | --- | --- | --- | --- | --- | --- | --- | --- | --- | --- | --- | --- |
| 1 | 10.00 | 2.00 | 0.00 | 0.00 | 0.00 | 0.00 | 10.00 | 0.00 | 2.00 | 0.00 | 0.00 | 0.00 | 2.42384E-20 | 2.8372E-06 |
| 2 | 10.00 | 2.00 | 0.00 | 0.00 | 0.00 | 0.00 | 10.00 | 0.00 | 2.00 | 0.00 | 0.00 | 0.00 | 2.42384E-20 | 3.9795E-06 |
| 3 | 10.00 | 2.00 | 0.00 | 0.00 | 0.00 | 0.00 | 10.00 | 0.00 | 2.00 | 0.00 | 0.00 | 0.00 | 2.42384E-20 | 3.8225E-06 |
| 4 | 10.00 | 2.00 | 0.00 | 0.00 | 0.00 | 0.00 | 10.00 | 0.00 | 2.00 | 0.00 | 0.00 | 0.00 | 9.53995E-22 | 3.3204E-06 |
| 5 | 10.00 | 2.00 | 0.00 | 0.00 | 0.00 | 0.00 | 10.00 | 0.00 | 2.00 | 0.00 | 0.00 | 0.00 | 8.29502E-19 | 3.5258E-06 |
| 6 | 10.00 | 2.00 | 0.00 | 0.00 | 0.00 | 0.00 | 10.00 | 0.00 | 2.00 | 0.00 | 0.00 | 0.00 | 2.03646E-20 | 3.5258E-06 |
| 7 | 10.00 | 2.00 | 0.00 | 0.00 | 0.00 | 0.00 | 10.00 | 0.00 | 2.00 | 0.00 | 0.00 | 0.00 | 2.12854E-21 | 3.2816E-06 |
| 8 | 10.00 | 2.00 | 0.00 | 0.00 | 0.00 | 0.00 | 10.00 | 0.00 | 2.00 | 0.00 | 0.00 | 0.00 | 3.42789E-19 | 0.00102828 |
| 9 | 10.00 | 2.00 | 0.00 | 0.00 | 0.00 | 0.00 | 10.00 | 0.00 | 2.00 | 0.00 | 0.00 | 0.00 | 2.63356E-20 | 3.4658E-06 |
| 10 | 10.00 | 2.00 | 0.00 | 0.00 | 0.00 | 0.00 | 10.00 | 0.00 | 2.00 | 0.00 | 0.00 | 0.00 | 4.65924E-20 | 6.9455E-06 |

Table 27 – Result of the 10 runs for the variable *X*_2_ of the 5-dimensional system with beta initial guesses uniformly distributed in the range [1, 10].

| run | $\alpha_{3}$ | $g_{31}$ | $g_{32}$ | $g_{33}$ | $g_{34}$ | $g_{35}$ | $\beta_{3}$ | $h_{31}$ | $h_{32}$ | $h_{33}$ | $h_{34}$ | $h_{35}$ | Error1 | Error2 |
| --- | --- | --- | --- | --- | --- | --- | --- | --- | --- | --- | --- | --- | --- | --- |
| 1 | 10.00 | 0.00 | -1.00 | 0.00 | 0.00 | 0.00 | 10.00 | 0.00 | -1.00 | 2.00 | 0.00 | 0.00 | 8.377E-19 | 1.003E-07 |
| 2 | 10.00 | 0.00 | -1.00 | 0.00 | 0.00 | 0.00 | 10.00 | 0.00 | -1.00 | 2.00 | 0.00 | 0.00 | 8.377E-19 | 9.063E-08 |
| 3 | 10.00 | 0.00 | -1.00 | 0.00 | 0.00 | 0.00 | 10.00 | 0.00 | -1.00 | 2.00 | 0.00 | 0.00 | 8.377E-19 | 8.256E-08 |
| 4 | 10.00 | 0.00 | -1.00 | 0.00 | 0.00 | 0.00 | 10.00 | 0.00 | -1.00 | 2.00 | 0.00 | 0.00 | 8.377E-19 | 8.798E-08 |
| 5 | 10.00 | 0.00 | -1.00 | 0.00 | 0.00 | 0.00 | 10.00 | 0.00 | -1.00 | 2.00 | 0.00 | 0.00 | 8.377E-19 | 1.094E-07 |
| 6 | 10.00 | 0.00 | -1.00 | 0.00 | 0.00 | 0.00 | 10.00 | 0.00 | -1.00 | 2.00 | 0.00 | 0.00 | 1.264E-18 | 1.094E-07 |
| 7 | 10.00 | 0.00 | -1.00 | 0.00 | 0.00 | 0.00 | 10.00 | 0.00 | -1.00 | 2.00 | 0.00 | 0.00 | 7.212E-19 | 8.733E-08 |
| 8 | 12.44 | 0.34 | -1.15 | -0.20 | -0.24 | 0.29 | 11.51 | -0.17 | -1.00 | -0.39 | 0.28 | 0.24 | 0.0004421 | 0.0278113 |
| 9 | 10.89 | 0.13 | -1.11 | -0.29 | -0.22 | 0.10 | 10.15 | -0.33 | -0.98 | -0.47 | 0.25 | 0.07 | 0.0004377 | 0.0003384 |
| 10 | 11.52 | -0.06 | -0.68 | -0.31 | -0.19 | 0.03 | 10.72 | -0.53 | -0.55 | -0.51 | 0.30 | 0.00 | 0.0004323 | 0.000172 |

Table 28 – Result of the 10 runs for the variable *X*_3_ of the 5-dimensional system with beta initial guesses uniformly distributed in the range [1, 10].

| Run | $\alpha_{4}$ | $g_{41}$ | $g_{42}$ | $g_{43}$ | $g_{44}$ | $g_{45}$ | $\beta_{4}$ | $h_{41}$ | $h_{42}$ | $h_{43}$ | $h_{44}$ | $h_{45}$ | Error1 | Error2 |
| --- | --- | --- | --- | --- | --- | --- | --- | --- | --- | --- | --- | --- | --- | --- |
| 1 | 6.44 | -0.63 | -0.12 | 0.83 | 0.15 | -1.41 | 12.00 | 1.92 | -1.00 | 0.90 | -0.26 | 0.39 | 0.0070596 | 5.196E-05 |
| 2 | 6.44 | -0.63 | -0.12 | 0.83 | 0.15 | -1.41 | 12.00 | 1.92 | -1.00 | 0.90 | -0.26 | 0.39 | 0.0070596 | 3.811E-05 |
| 3 | 6.44 | -0.63 | -0.12 | 0.83 | 0.15 | -1.41 | 12.00 | 1.92 | -1.00 | 0.90 | -0.26 | 0.39 | 0.0070596 | 5.728E-05 |
| 4 | 6.44 | -0.63 | -0.12 | 0.83 | 0.15 | -1.41 | 12.00 | 1.92 | -1.00 | 0.90 | -0.26 | 0.39 | 0.0070596 | 3.681E-05 |
| 5 | 6.44 | -0.63 | -0.12 | 0.83 | 0.15 | -1.41 | 12.00 | 1.92 | -1.00 | 0.90 | -0.26 | 0.39 | 0.0070596 | 4.64E-05 |
| 6 | 6.44 | -0.63 | -0.12 | 0.83 | 0.15 | -1.41 | 12.00 | 1.92 | -1.00 | 0.90 | -0.26 | 0.39 | 0.0070596 | 4.64E-05 |
| 7 | 6.44 | -0.63 | -0.12 | 0.83 | 0.15 | -1.41 | 12.00 | 1.92 | -1.00 | 0.90 | -0.26 | 0.39 | 0.0070596 | 3.872E-05 |
| 8 | 6.25 | -0.75 | -0.05 | 0.53 | 0.21 | -1.45 | 12.00 | 1.98 | -1.00 | 0.70 | -0.33 | 0.42 | 0.0079995 | 0.0083868 |
| 9 | 6.60 | -0.63 | -0.16 | 0.90 | 0.15 | -1.38 | 12.00 | 1.81 | -1.00 | 0.93 | -0.20 | 0.35 | 0.0073492 | 0.0003581 |
| 10 | 6.45 | -0.63 | -0.12 | 0.80 | 0.15 | -1.40 | 12.00 | 1.91 | -1.00 | 0.88 | -0.27 | 0.38 | 0.0074822 | 5.074E-05 |

Table 29 – Result of the 10 runs for the variable *X*_4_ of the 5-dimensional system with beta initial guesses uniformly distributed in the range [1, 10].

| run | $\alpha_{5}$ | $g_{51}$ | $g_{52}$ | $g_{53}$ | $g_{54}$ | $g_{55}$ | $\beta_{5}$ | $h_{51}$ | $h_{52}$ | $h_{53}$ | $h_{54}$ | $h_{55}$ | Error1 | Error2 |
| --- | --- | --- | --- | --- | --- | --- | --- | --- | --- | --- | --- | --- | --- | --- |
| 1 | 13.25 | 0.61 | 0.67 | 1.36 | 1.45 | -0.05 | 11.99 | 0.01 | 0.85 | -0.01 | 0.02 | 1.72 | 0.0006523 | 2.438E-06 |
| 2 | 10.58 | -0.27 | 0.61 | -0.56 | 2.11 | -0.17 | 10.50 | -0.29 | 0.64 | -0.09 | 0.06 | 1.75 | 4.939E-07 | 7.784E-06 |
| 3 | 14.33 | 1.76 | -0.06 | 1.71 | 1.08 | 0.17 | 11.27 | 0.31 | 0.53 | 0.00 | 0.00 | 1.62 | 0.0015222 | 4.445E-06 |
| 4 | 10.38 | 0.08 | 0.20 | 0.60 | 1.86 | -0.06 | 10.09 | -0.09 | 0.25 | 0.00 | 0.00 | 1.96 | 1.997E-05 | 4.987E-06 |
| 5 | 11.87 | 0.56 | 0.00 | -0.31 | 1.80 | 0.09 | 11.17 | 0.25 | 0.16 | -0.03 | 0.02 | 1.78 | 0.000263 | 6.627E-06 |
| 6 | 11.87 | 0.56 | 0.00 | -0.31 | 1.80 | 0.09 | 11.17 | 0.25 | 0.16 | -0.03 | 0.02 | 1.78 | 0.000263 | 6.627E-06 |
| 7 | 10.82 | 0.19 | 0.27 | 0.68 | 1.79 | -0.05 | 10.39 | -0.05 | 0.34 | 0.00 | 0.00 | 1.91 | 4.827E-05 | 4.707E-06 |
| 8 | 11.25 | 0.21 | 0.15 | -0.20 | 1.90 | 0.04 | 11.04 | 0.11 | 0.19 | -0.02 | 0.02 | 1.87 | 9.559E-05 | 0.0045021 |
| 9 | 13.75 | 1.48 | 0.02 | 1.84 | 1.15 | 0.14 | 11.23 | 0.26 | 0.49 | 0.00 | 0.00 | 1.71 | 0.0008662 | 2.153E-05 |
| 10 | 10.50 | -0.24 | 0.53 | -0.53 | 2.10 | -0.14 | 10.45 | -0.25 | 0.55 | -0.08 | 0.05 | 1.78 | 3.058E-07 | 3.996E-05 |

Table 30 – Result of the 10 runs for the variable *X*_5_ of the 5-dimensional system with beta initial guesses uniformly distributed in the range [1, 10].

## 2-Dimensional system results – noise-free time series

Tables 31-32 show the parameters found with the proposed algorithm for the 2-dimensional system (Equation 1) using the first set of initial values of the Table 3.

| run | $\alpha_{1}$ | $g_{11}$ | $g_{12}$ | $\beta_{1}$ | $h_{11}$ | $h_{12}$ | Error1 | Error2 |
| --- | --- | --- | --- | --- | --- | --- | --- | --- |
| 1 | 3.00 | 0.00 | -2.00 | 1.00 | 0.50 | 1.00 | 4.96082E-18 | 0.13826495 |
| 2 | 8.51 | 0.19 | -0.84 | 6.61 | 0.31 | -0.15 | 3.28642E-10 | 0.13826782 |
| 3 | 8.61 | 0.19 | -0.83 | 6.71 | 0.31 | -0.15 | 3.29596E-10 | 0.13826787 |
| 4 | 5.91 | 0.16 | -1.03 | 4.00 | 0.34 | 0.04 | 2.79763E-10 | 0.13826757 |
| 5 | 9.79 | 0.20 | -0.79 | 7.89 | 0.30 | -0.20 | 3.40998E-10 | 0.13826792 |
| 6 | 5.91 | 0.16 | -1.03 | 4.00 | 0.34 | 0.04 | 2.79756E-10 | 0.13826747 |
| 7 | 5.91 | 0.16 | -1.03 | 4.00 | 0.34 | 0.04 | 2.79647E-10 | 0.13826764 |
| 8 | 5.91 | 0.16 | -1.03 | 4.00 | 0.34 | 0.04 | 2.79763E-10 | 0.13826761 |
| 9 | 5.91 | 0.16 | -1.03 | 4.00 | 0.34 | 0.04 | 2.79723E-10 | 0.13826759 |
| 10 | 3.00 | 0.00 | -2.00 | 1.00 | 0.50 | 1.00 | 9.07017E-16 | 0.13826492 |

Table 31 – Result of the 10 runs for the variable *X*_1_ of the 2-dimensional system with beta initial guesses randomly distributed in the range [0.1, 8].

| run | $\alpha_{2}$ | $g_{21}$ | $g_{22}$ | $\beta_{2}$ | $h_{21}$ | $h_{22}$ | Error1 | Error2 |
| --- | --- | --- | --- | --- | --- | --- | --- | --- |
| 1 | 3.00 | 0.00 | -2.00 | 1.00 | 0.50 | 1.00 | 4.96082E-18 | 0.13826495 |
| 2 | 8.51 | 0.19 | -0.84 | 6.61 | 0.31 | -0.15 | 3.28642E-10 | 0.13826782 |
| 3 | 8.61 | 0.19 | -0.83 | 6.71 | 0.31 | -0.15 | 3.29596E-10 | 0.13826787 |
| 4 | 5.91 | 0.16 | -1.03 | 4.00 | 0.34 | 0.04 | 2.79763E-10 | 0.13826757 |
| 5 | 9.79 | 0.20 | -0.79 | 7.89 | 0.30 | -0.20 | 3.40998E-10 | 0.13826792 |
| 6 | 5.91 | 0.16 | -1.03 | 4.00 | 0.34 | 0.04 | 2.79756E-10 | 0.13826747 |
| 7 | 5.91 | 0.16 | -1.03 | 4.00 | 0.34 | 0.04 | 2.79647E-10 | 0.13826764 |
| 8 | 5.91 | 0.16 | -1.03 | 4.00 | 0.34 | 0.04 | 2.79763E-10 | 0.13826761 |
| 9 | 5.91 | 0.16 | -1.03 | 4.00 | 0.34 | 0.04 | 2.79723E-10 | 0.13826759 |
| 10 | 3.00 | 0.00 | -2.00 | 1.00 | 0.50 | 1.00 | 9.07017E-16 | 0.13826492 |

Table 32 – Result of the 10 runs for the variable *X*_2_ of the 2-dimensional system with beta initial guesses randomly distributed in the range [0.1, 8].

## 10-Dimension system result – noise-free

Figure 7 shows the result of the algorithm with a 10-dimensional system (Equation 6).

Figure 7 - Dynamical result of the integrated system (full lines) found by the proposed algorithm.

## 2- and 4-dimensional rescaled system results – noise-free time series

The following results were obtained using the rescaled 2 and 4-dimensional systems (Equations 4 and 5 respectively).

**2-Dimensional system**

| run | $\alpha_{1}$ | $g_{11}$ | $g_{12}$ | $\beta_{1}$ | $h_{11}$ | $h_{12}$ | Error1 | Error2 |
| --- | --- | --- | --- | --- | --- | --- | --- | --- |
| 1 | 12.00 | 0.00 | -2.00 | 4.00 | 0.50 | 1.00 | 5.71139E-18 | 0.00039181 |
| 2 | 12.00 | 0.00 | -2.00 | 4.00 | 0.50 | 1.00 | 6.68559E-19 | 0.00030513 |
| 3 | 12.00 | 0.00 | -2.00 | 4.00 | 0.50 | 1.00 | 1.8914E-18 | 0.00030513 |
| 4 | 12.00 | 0.00 | -2.00 | 4.00 | 0.50 | 1.00 | 1.20093E-18 | 0.00030513 |
| 5 | 12.00 | 0.00 | -2.00 | 4.00 | 0.50 | 1.00 | 2.02577E-19 | 0.00030513 |
| 6 | 12.00 | 0.00 | -2.00 | 4.00 | 0.50 | 1.00 | 4.32945E-20 | 0.00030513 |
| 7 | 12.00 | 0.00 | -2.00 | 4.00 | 0.50 | 1.00 | 3.49081E-18 | 0.00030513 |
| 8 | 12.00 | 0.00 | -2.00 | 4.00 | 0.50 | 1.00 | 8.02436E-20 | 0.00030513 |
| 9 | 12.00 | 0.00 | -2.00 | 4.00 | 0.50 | 1.00 | 1.46175E-19 | 0.00030513 |
| 10 | 12.00 | 0.00 | -2.00 | 4.00 | 0.50 | 1.00 | 1.00616E-18 | 0.00038688 |

Table 33 – Result of the 10 runs for the variable *X*_1_ of the rescaled 2-dimensional system with beta initial guesses randomly distributed in the range [0.1, 10] and data set 3 of the table 3.

| run | $\alpha_{2}$ | $g_{21}$ | $g_{22}$ | $\beta_{2}$ | $h_{21}$ | $h_{22}$ | Error1 | Error2 |
| --- | --- | --- | --- | --- | --- | --- | --- | --- |
| 1 | 8.00 | 0.38 | 0.87 | 8.00 | 0.13 | 0.62 | 4.42263E-05 | 0.00016116 |
| 2 | 4.00 | 0.50 | 1.00 | 4.00 | 0.00 | 0.50 | 4.76375E-19 | 0.00012111 |
| 3 | 4.00 | 0.50 | 1.00 | 4.00 | 0.00 | 0.50 | 7.93078E-19 | 0.00012111 |
| 4 | 4.00 | 0.50 | 1.00 | 4.00 | 0.00 | 0.50 | 2.14745E-18 | 0.00012111 |
| 5 | 4.00 | 0.50 | 1.00 | 4.00 | 0.00 | 0.50 | 4.01564E-19 | 0.00012111 |
| 6 | 4.00 | 0.50 | 1.00 | 4.00 | 0.00 | 0.50 | 7.04372E-19 | 0.00012111 |
| 7 | 4.00 | 0.50 | 1.00 | 4.00 | 0.00 | 0.50 | 1.41675E-18 | 0.00012111 |
| 8 | 4.00 | 0.50 | 1.00 | 4.00 | 0.00 | 0.50 | 3.26273E-19 | 0.00012111 |
| 9 | 4.00 | 0.50 | 1.00 | 4.00 | 0.00 | 0.50 | 4.68332E-19 | 0.00012111 |
| 10 | 8.00 | 0.38 | 0.88 | 8.00 | 0.13 | 0.63 | 4.44377E-05 | 0.00015885 |

Table 34 – Result of the 10 runs for the variable *X*_2_ of the rescaled 2-dimensional system with beta initial guesses randomly distributed in the range [0.1, 10] and data set 2 of the table 3.

| run | $\alpha_{1}$ | $g_{11}$ | $g_{12}$ | $\beta_{1}$ | $h_{11}$ | $h_{12}$ | Error1 | Error2 |
| --- | --- | --- | --- | --- | --- | --- | --- | --- |
| 1 | 12.00 | 0.00 | -2.00 | 4.00 | 0.50 | 1.00 | 2.11945E-19 | 9.16738E-05 |
| 2 | 12.00 | 0.00 | -2.00 | 4.00 | 0.50 | 1.00 | 1.54089E-19 | 9.16738E-05 |
| 3 | 12.00 | 0.00 | -2.00 | 4.00 | 0.50 | 1.00 | 1.235E-18 | 9.16738E-05 |
| 4 | 12.00 | 0.00 | -2.00 | 4.00 | 0.50 | 1.00 | 9.16002E-19 | 9.16738E-05 |
| 5 | 12.00 | 0.00 | -2.00 | 4.00 | 0.50 | 1.00 | 4.71097E-20 | 9.16738E-05 |
| 6 | 12.00 | 0.00 | -2.00 | 4.00 | 0.50 | 1.00 | 5.44856E-19 | 9.16738E-05 |
| 7 | 12.00 | 0.00 | -2.00 | 4.00 | 0.50 | 1.00 | 1.75402E-18 | 9.16738E-05 |
| 8 | 12.00 | 0.00 | -2.00 | 4.00 | 0.50 | 1.00 | 1.66197E-19 | 9.17383E-05 |
| 9 | 12.00 | 0.00 | -2.00 | 4.00 | 0.50 | 1.00 | 8.54714E-19 | 9.17351E-05 |
| 10 | 12.00 | 0.00 | -2.00 | 4.00 | 0.50 | 1.00 | 2.73572E-19 | 9.17323E-05 |

Table 35 – Result of the 10 runs for the variable *X*_1_ of the rescaled 2-dimensional system with beta initial guesses randomly distributed in the range [0.1, 10] and data set 3 of the table 3.

| run | $\alpha_{2}$ | $g_{21}$ | $g_{22}$ | $\beta_{2}$ | $h_{21}$ | $h_{22}$ | Error1 | Error2 |
| --- | --- | --- | --- | --- | --- | --- | --- | --- |
| 1 | 4.00 | 0.50 | 1.00 | 4.00 | 0.00 | 0.50 | 8.65611E-20 | 3.6821E-05 |
| 2 | 4.00 | 0.50 | 1.00 | 4.00 | 0.00 | 0.50 | 2.70191E-19 | 3.6821E-05 |
| 3 | 4.00 | 0.50 | 1.00 | 4.00 | 0.00 | 0.50 | 1.76209E-20 | 3.6821E-05 |
| 4 | 4.00 | 0.50 | 1.00 | 4.00 | 0.00 | 0.50 | 3.7232E-21 | 3.6821E-05 |
| 5 | 4.00 | 0.50 | 1.00 | 4.00 | 0.00 | 0.50 | 3.37995E-20 | 3.6821E-05 |
| 6 | 4.00 | 0.50 | 1.00 | 4.00 | 0.00 | 0.50 | 1.4726E-21 | 3.6821E-05 |
| 7 | 4.00 | 0.50 | 1.00 | 4.00 | 0.00 | 0.50 | 9.99782E-20 | 3.6821E-05 |
| 8 | 8.00 | 0.38 | 0.88 | 8.00 | 0.13 | 0.63 | 2.82734E-10 | 3.6854E-05 |
| 9 | 7.44 | 0.38 | 0.88 | 7.44 | 0.12 | 0.62 | 2.54172E-10 | 3.6852E-05 |
| 10 | 7.05 | 0.39 | 0.89 | 7.05 | 0.11 | 0.61 | 2.31114E-10 | 3.6851E-05 |

Table 36 – Result of the 10 runs for the variable *X*_2_ of the rescaled 2-dimensional system with beta initial guesses randomly distributed in the range [0.1, 10] and data set 3 of the table 3.

**4-Dimensional system**

| run | $\alpha_{1}$ | $g_{11}$ | $g_{12}$ | $g_{13}$ | $g_{14}$ | $\beta_{1}$ | $h_{11}$ | $h_{12}$ | $h_{13}$ | $h_{14}$ | Error1 | Error2 |
| --- | --- | --- | --- | --- | --- | --- | --- | --- | --- | --- | --- | --- |
| 1 | 12 | 0.00 | 0.00 | -0.80 | 0.00 | 10.00 | 0.50 | 0.00 | 0.00 | 0.00 | 1.7967E-17 | 3.42886E-05 |
| 2 | 11.99 | 0.00 | 0.00 | -0.80 | 0.00 | 10.00 | 0.50 | 0.00 | 0.00 | 0.00 | 1.4559E-18 | 3.42886E-05 |
| 3 | 12 | 0.00 | 0.00 | -0.80 | 0.00 | 10.00 | 0.50 | 0.00 | 0.00 | 0.00 | 1.6562E-20 | 3.42886E-05 |
| 4 | 12 | 0.00 | 0.00 | -0.80 | 0.00 | 10.00 | 0.50 | 0.00 | 0.00 | 0.00 | 3.4834E-19 | 3.42886E-05 |
| 5 | 12 | 0.00 | 0.00 | -0.80 | 0.00 | 10.00 | 0.50 | 0.00 | 0.00 | 0.00 | 2.539E-18 | 3.42886E-05 |
| 6 | 12 | 0.00 | 0.00 | -0.80 | 0.00 | 10.00 | 0.50 | 0.00 | 0.00 | 0.00 | 2.1632E-19 | 3.42886E-05 |
| 7 | 11.99 | 0.00 | 0.00 | -0.80 | 0.00 | 10.00 | 0.50 | 0.00 | 0.00 | 0.00 | 1.636E-19 | 3.42886E-05 |
| 8 | 12 | 0.00 | 0.00 | -0.80 | 0.00 | 10.00 | 0.50 | 0.00 | 0.00 | 0.00 | 3.3202E-19 | 3.42886E-05 |
| 9 | 12 | 0.00 | 0.00 | -0.80 | 0.00 | 10.00 | 0.50 | 0.00 | 0.00 | 0.00 | 1.9844E-18 | 3.42886E-05 |
| 10 | 12 | 0.00 | 0.00 | -0.80 | 0.00 | 10.00 | 0.50 | 0.00 | 0.00 | 0.00 | 1.9287E-19 | 3.42886E-05 |

Table 37 – Result of the 10 runs for the variable *X*_1_ of the rescaled 4-dimensional system with beta initial guesses randomly distributed in the range [1, 12] and data set 3 of the table 1.

.

| run | $\alpha_{2}$ | $g_{21}$ | $g_{22}$ | $g_{23}$ | $g_{24}$ | $\beta_{2}$ | $h_{21}$ | $h_{22}$ | $h_{23}$ | $h_{24}$ | Error1 | Error2 |
| --- | --- | --- | --- | --- | --- | --- | --- | --- | --- | --- | --- | --- |
| 1 | 16.00 | 0.50 | 0.00 | 0.00 | 0.00 | 6.00 | 0.00 | 0.75 | 0.00 | 0.00 | 1.0857E-18 | 7.05026E-05 |
| 2 | 16.00 | 0.50 | 0.00 | 0.00 | 0.00 | 6.00 | 0.00 | 0.75 | 0.00 | 0.00 | 1.12089E-20 | 7.05026E-05 |
| 3 | 16.00 | 0.50 | 0.00 | 0.00 | 0.00 | 6.00 | 0.00 | 0.75 | 0.00 | 0.00 | 4.2539E-19 | 7.05026E-05 |
| 4 | 16.00 | 0.50 | 0.00 | 0.00 | 0.00 | 6.00 | 0.00 | 0.75 | 0.00 | 0.00 | 3.48427E-19 | 7.05026E-05 |
| 5 | 16.00 | 0.50 | 0.00 | 0.00 | 0.00 | 6.00 | 0.00 | 0.75 | 0.00 | 0.00 | 4.1347E-19 | 7.05026E-05 |
| 6 | 16.00 | 0.50 | 0.00 | 0.00 | 0.00 | 6.00 | 0.00 | 0.75 | 0.00 | 0.00 | 7.55033E-19 | 7.05026E-05 |
| 7 | 16.00 | 0.50 | 0.00 | 0.00 | 0.00 | 6.00 | 0.00 | 0.75 | 0.00 | 0.00 | 1.65911E-18 | 7.05026E-05 |
| 8 | 16.00 | 0.50 | 0.00 | 0.00 | 0.00 | 6.00 | 0.00 | 0.75 | 0.00 | 0.00 | 5.68747E-19 | 7.05026E-05 |
| 9 | 16.00 | 0.50 | 0.00 | 0.00 | 0.00 | 6.00 | 0.00 | 0.75 | 0.00 | 0.00 | 1.78691E-19 | 7.05026E-05 |
| 10 | 16.00 | 0.50 | 0.00 | 0.00 | 0.00 | 6.00 | 0.00 | 0.75 | 0.00 | 0.00 | 9.89292E-20 | 7.05026E-05 |

Table 38 – Result of the 10 runs for the variable *X*_2_ of the rescaled 4-dimensional system with beta initial guesses randomly distributed in the range [1, 12] and data set 3 of the table 1.

| run | $\alpha_{3}$ | $g_{31}$ | $g_{32}$ | $g_{33}$ | $g_{34}$ | $\beta_{3}$ | $h_{31}$ | $h_{32}$ | $h_{33}$ | $h_{34}$ | Error1 | Error2 |
| --- | --- | --- | --- | --- | --- | --- | --- | --- | --- | --- | --- | --- |
| 1 | 6.00 | 0.00 | 0.75 | 0.00 | 0.00 | 10.00 | 0.00 | 0.00 | 0.50 | 0.20 | 1.99232E-18 | 6.12698E-05 |
| 2 | 6.00 | 0.00 | 0.75 | 0.00 | 0.00 | 10.00 | 0.00 | 0.00 | 0.50 | 0.20 | 2.72158E-18 | 6.12698E-05 |
| 3 | 6.00 | 0.00 | 0.75 | 0.00 | 0.00 | 10.00 | 0.00 | 0.00 | 0.50 | 0.20 | 2.01479E-19 | 6.12698E-05 |
| 4 | 6.00 | 0.00 | 0.75 | 0.00 | 0.00 | 10.00 | 0.00 | 0.00 | 0.50 | 0.20 | 1.07911E-19 | 6.12698E-05 |
| 5 | 6.00 | 0.00 | 0.75 | 0.00 | 0.00 | 10.00 | 0.00 | 0.00 | 0.50 | 0.20 | 1.43103E-18 | 6.12698E-05 |
| 6 | 6.00 | 0.00 | 0.75 | 0.00 | 0.00 | 10.00 | 0.00 | 0.00 | 0.50 | 0.20 | 2.42209E-19 | 6.12698E-05 |
| 7 | 6.00 | 0.00 | 0.75 | 0.00 | 0.00 | 10.00 | 0.00 | 0.00 | 0.50 | 0.20 | 7.16605E-19 | 6.12698E-05 |
| 8 | 6.00 | 0.00 | 0.75 | 0.00 | 0.00 | 10.00 | 0.00 | 0.00 | 0.50 | 0.20 | 8.18388E-19 | 6.12698E-05 |
| 9 | 6.00 | 0.00 | 0.75 | 0.00 | 0.00 | 10.00 | 0.00 | 0.00 | 0.50 | 0.20 | 5.54136E-19 | 6.12698E-05 |
| 10 | 6.00 | 0.00 | 0.75 | 0.00 | 0.00 | 10.00 | 0.00 | 0.00 | 0.50 | 0.20 | 4.17374E-18 | 6.12698E-05 |

Table 39 – Result of the 10 runs for the variable *X*_3_ of the rescaled 4-dimensional system with beta initial guesses randomly distributed in the range [1, 12] and data set 3 of the table 1.

| run | $\alpha_{4}$ | $g_{41}$ | $g_{42}$ | $g_{43}$ | $g_{44}$ | $\beta_{4}$ | $h_{41}$ | $h_{42}$ | $h_{43}$ | $h_{44}$ | Error1 | Error2 |
| --- | --- | --- | --- | --- | --- | --- | --- | --- | --- | --- | --- | --- |
| 1 | 4.00 | 0.50 | 0.00 | 0.00 | 0.00 | 12.00 | 0.00 | 0.00 | 0.00 | 0.80 | 7.11853E-21 | 7.70916E-07 |
| 2 | 4.00 | 0.50 | 0.00 | 0.00 | 0.00 | 12.00 | 0.00 | 0.00 | 0.00 | 0.80 | 5.86408E-20 | 7.70916E-07 |
| 3 | 4.00 | 0.50 | 0.00 | 0.00 | 0.00 | 12.00 | 0.00 | 0.00 | 0.00 | 0.80 | 5.15877E-22 | 7.70916E-07 |
| 4 | 4.00 | 0.50 | 0.00 | 0.00 | 0.00 | 12.00 | 0.00 | 0.00 | 0.00 | 0.80 | 3.31997E-19 | 7.70916E-07 |
| 5 | 4.00 | 0.50 | 0.00 | 0.00 | 0.00 | 12.00 | 0.00 | 0.00 | 0.00 | 0.80 | 3.67002E-21 | 7.70916E-07 |
| 6 | 4.00 | 0.50 | 0.00 | 0.00 | 0.00 | 12.00 | 0.00 | 0.00 | 0.00 | 0.80 | 3.40673E-21 | 7.70916E-07 |
| 7 | 4.00 | 0.50 | 0.00 | 0.00 | 0.00 | 12.00 | 0.00 | 0.00 | 0.00 | 0.80 | 3.15828E-21 | 7.70916E-07 |
| 8 | 4.00 | 0.50 | 0.00 | 0.00 | 0.00 | 12.00 | 0.00 | 0.00 | 0.00 | 0.80 | 5.02213E-21 | 7.70916E-07 |
| 9 | 4.00 | 0.50 | 0.00 | 0.00 | 0.00 | 12.00 | 0.00 | 0.00 | 0.00 | 0.80 | 1.09829E-21 | 7.70916E-07 |
| 10 | 4.00 | 0.50 | 0.00 | 0.00 | 0.00 | 12.00 | 0.00 | 0.00 | 0.00 | 0.80 | 6.02689E-19 | 7.70916E-07 |

Table 40 – Result of the 10 runs for the variable *X*_4_ of the rescaled 4-dimensional system with beta initial guesses randomly distributed in the range [1, 12] and data set 3 of the table 1.

## 4-Dimensional system results – noisy time series

Tables 41-44 show the parameters found with the proposed algorithm for the 4-dimensional system (Equation 2) using noisy time series.

| run | $\alpha_{1}$ | $g_{11}$ | $g_{12}$ | $g_{13}$ | $g_{14}$ | $\beta_{1}$ | $h_{11}$ | $h_{12}$ | $h_{13}$ | $h_{14}$ | Error1 | Error2 |
| --- | --- | --- | --- | --- | --- | --- | --- | --- | --- | --- | --- | --- |
| 1 | 10.38 | 0.53 | 1.07 | -0.61 | -0.62 | 9.49 | 0.61 | 1.11 | -0.53 | -0.67 | 0.4762953 | 0.3589214 |
| 2 | 10.04 | 0.53 | 1.07 | -0.60 | -0.62 | 9.15 | 0.61 | 1.10 | -0.52 | -0.66 | 0.4754111 | 0.3621649 |
| 3 | 7.49 | 0.53 | 1.05 | -0.43 | -0.44 | 6.50 | 0.66 | 1.10 | -0.32 | -0.51 | 0.4120744 | 0.212672 |
| 4 | 7.27 | 0.52 | 1.02 | -0.44 | -0.43 | 6.27 | 0.65 | 1.08 | -0.31 | -0.50 | 0.4005951 | 0.2729454 |
| 5 | 6.79 | 0.55 | 0.97 | -0.49 | -0.56 | 5.83 | 0.69 | 1.03 | -0.36 | -0.63 | 0.4618373 | 0.321666 |
| 6 | 3.80 | 0.59 | 0.61 | -0.10 | -0.32 | 2.57 | 0.96 | 0.76 | 0.28 | -0.49 | 0.4082135 | 0.1781068 |
| 7 | 10.97 | 0.52 | 1.08 | -0.62 | -0.63 | 10.08 | 0.60 | 1.12 | -0.55 | -0.67 | 0.477732 | 0.328733 |
| 8 | 9.85 | 0.53 | 1.06 | -0.60 | -0.62 | 8.95 | 0.61 | 1.10 | -0.52 | -0.66 | 0.474871 | 0.244898 |
| 9 | 10.02 | 0.53 | 1.07 | -0.60 | -0.62 | 9.12 | 0.61 | 1.10 | -0.52 | -0.66 | 0.475339 | 0.348363 |
| 10 | 9.24 | 0.53 | 1.05 | -0.58 | -0.61 | 8.33 | 0.62 | 1.09 | -0.49 | -0.66 | 0.473011 | 0.325423 |

Table 41 – Result of the 10 runs for the variable *X*_1_ of the 4-dimensional system with beta initial guesses randomly distributed in the range [1, 12].

| run | $\alpha_{2}$ | $g_{21}$ | $g_{22}$ | $g_{23}$ | $g_{24}$ | $\beta_{2}$ | $h_{21}$ | $h_{22}$ | $h_{23}$ | $h_{24}$ | Error1 | Error2 |
| --- | --- | --- | --- | --- | --- | --- | --- | --- | --- | --- | --- | --- |
| 1 | 16.87 | 0.09 | 0.19 | 0.00 | -0.12 | 11.77 | 0.12 | 0.32 | 0.17 | -0.20 | 0.4738779 | 2.5140256 |
| 2 | 16.03 | 0.09 | 0.18 | -0.01 | -0.12 | 10.95 | 0.12 | 0.33 | 0.17 | -0.21 | 0.4708193 | 3.0556242 |
| 3 | 7.68 | -0.16 | 0.39 | -0.09 | 0.28 | 2.35 | 0.00 | 0.78 | 0.55 | 0.00 | 0.5107566 | 2.1002638 |
| 4 | 12.04 | 0.06 | 0.20 | -0.03 | -0.09 | 6.96 | 0.12 | 0.39 | 0.24 | -0.23 | 0.4966603 | 1.6639319 |
| 5 | 9.20 | 0.07 | 0.25 | -0.10 | -0.04 | 4.27 | 0.13 | 0.54 | 0.24 | -0.22 | 0.4698792 | 2.4678547 |
| 6 | 7.67 | -0.06 | 0.42 | -0.11 | 0.06 | 2.83 | 0.11 | 0.74 | 0.34 | -0.23 | 0.5198797 | 1.1053894 |
| 7 | 10.05 | 0.13 | 0.25 | -0.03 | -0.08 | 5.12 | 0.19 | 0.51 | 0.25 | -0.24 | 0.481542 | 2.284232 |
| 8 | 8.07 | -0.13 | 0.55 | -0.09 | 0.08 | 3.30 | 0.06 | 0.82 | 0.33 | -0.20 | 0.5677575 | 1.7613932 |
| 9 | 17.05 | 0.09 | 0.19 | 0.00 | -0.12 | 11.95 | 0.12 | 0.32 | 0.17 | -0.20 | 0.4746868 | 2.6943604 |
| 10 | 13.51 | 0.16 | 0.16 | 0.03 | -0.17 | 8.47 | 0.21 | 0.33 | 0.23 | -0.29 | 0.4833689 | 1.7781254 |

Table 42 – Result of the 10 runs for the variable *X*_2_ of the 4-dimensional system with beta initial guesses randomly distributed in the range [1, 12].

| run | $\alpha_{3}$ | $g_{31}$ | $g_{32}$ | $g_{33}$ | $g_{34}$ | $\beta_{3}$ | $h_{31}$ | $h_{32}$ | $h_{33}$ | $h_{34}$ | Error1 | Error2 |
| --- | --- | --- | --- | --- | --- | --- | --- | --- | --- | --- | --- | --- |
| 1 | 5.84 | 0.40 | 0.50 | 0.33 | -0.60 | 5.50 | 0.39 | 0.41 | 0.32 | -0.66 | 0.2020232 | 4.0742276 |
| 2 | 3.45 | 0.44 | 0.44 | 0.39 | -0.57 | 3.09 | 0.41 | 0.28 | 0.36 | -0.69 | 0.2055951 | 5.705558 |
| 3 | 8.55 | 0.37 | 0.56 | 0.34 | -0.62 | 8.21 | 0.36 | 0.51 | 0.33 | -0.66 | 0.2160534 | 2.6206851 |
| 4 | 4.23 | 0.10 | 0.70 | 0.37 | -0.18 | 3.96 | 0.07 | 0.53 | 0.39 | -0.26 | 0.2222057 | 2.2206794 |
| 5 | 4.67 | 0.41 | 0.48 | 0.35 | -0.59 | 4.32 | 0.40 | 0.37 | 0.34 | -0.67 | 0.2030091 | 4.7700925 |
| 6 | 9.33 | 0.37 | 0.56 | 0.33 | -0.62 | 8.99 | 0.36 | 0.51 | 0.33 | -0.66 | 0.2156485 | 0.730108 |
| 7 | 4.84 | 0.41 | 0.48 | 0.35 | -0.59 | 4.49 | 0.39 | 0.37 | 0.33 | -0.67 | 0.2028097 | 4.8496191 |
| 8 | 10.35 | 0.36 | 0.57 | 0.33 | -0.62 | 10.01 | 0.36 | 0.52 | 0.33 | -0.65 | 0.2152312 | 3.1622848 |
| 9 | 4.46 | 0.06 | 0.63 | 0.10 | -0.22 | 4.21 | 0.00 | 0.44 | 0.06 | -0.31 | 0.2010062 | 4.7682708 |
| 10 | 4.58 | 0.02 | 0.85 | 0.01 | -0.42 | 4.29 | 0.00 | 0.74 | 0.00 | -0.49 | 0.2204152 | 2.301361 |

Table 43 – Result of the 10 runs for the variable *X*_3_ of the 4-dimensional system with beta initial guesses randomly distributed in the range [1, 12].

| run | $\alpha_{4}$ | $g_{41}$ | $g_{42}$ | $g_{43}$ | $g_{44}$ | $\beta_{4}$ | $h_{41}$ | $h_{42}$ | $h_{43}$ | $h_{44}$ | Error1 | Error2 |
| --- | --- | --- | --- | --- | --- | --- | --- | --- | --- | --- | --- | --- |
| 1 | 5.48 | 0.85 | 0.47 | -0.95 | -0.71 | 6.56 | 0.72 | 0.50 | -1.00 | -0.56 | 0.0291203 | 0.4771414 |
| 2 | 5.49 | 0.86 | 0.49 | -0.95 | -0.69 | 6.57 | 0.73 | 0.53 | -1.00 | -0.55 | 0.0291989 | 0.4827924 |
| 3 | 10.74 | 0.76 | 0.63 | -0.97 | -0.54 | 11.81 | 0.69 | 0.65 | -1.00 | -0.46 | 0.027442 | 0.4236371 |
| 4 | 6.56 | 0.81 | 0.59 | -0.95 | -0.58 | 7.63 | 0.69 | 0.62 | -1.00 | -0.46 | 0.0275361 | 0.4594413 |
| 5 | 9.68 | 0.50 | 0.48 | -0.96 | -0.43 | 10.92 | 0.42 | 0.50 | -1.00 | -0.33 | 0.0158422 | 0.44427 |
| 6 | 10.16 | 0.44 | 0.84 | -0.89 | -0.34 | 11.30 | 0.36 | 0.86 | -0.92 | -0.26 | 0.0269737 | 0.3826479 |
| 7 | 5.81 | 0.82 | 0.56 | -0.95 | -0.62 | 6.89 | 0.70 | 0.60 | -1.00 | -0.48 | 0.0278796 | 0.4574748 |
| 8 | 6.35 | 0.81 | 0.58 | -0.95 | -0.59 | 7.42 | 0.69 | 0.62 | -1.00 | -0.46 | 0.0274093 | 0.4284966 |
| 9 | 8.65 | 0.79 | 0.61 | -0.96 | -0.56 | 9.72 | 0.70 | 0.63 | -1.00 | -0.46 | 0.0274775 | 0.4720405 |
| 10 | 7.91 | 0.79 | 0.60 | -0.96 | -0.56 | 8.98 | 0.70 | 0.63 | -1.00 | -0.46 | 0.0273709 | 0.4441718 |

Table 44 – Result of the 10 runs for the variable *X*_4_ of the 4-dimensional system with beta initial guesses randomly distributed in the range [1, 12].

## 3-Dimensional linear pathway system – noise-free time series.

In order to test the constrained version of the proposed algorithm, we performed tests on a 3-dimensional linear pathway system (see Figure 8 in the main manuscript). The test results are shown in Table 45

|  | *α* | $g_{i1}$ | $g_{i2}$ | $g_{i3}$ | *β* | $h_{i1}$ | $h_{i2}$ | $h_{i3}$ | sum of squared errors |
| --- | --- | --- | --- | --- | --- | --- | --- | --- | --- |
| *X_1_* | 12.00 | 0 | 0 | -0.8 | 10.0 | 0.5 | 0 | 0 | 9.6e-15 |
| *X_2_* | 10.0 | 0.5 | 0 | 0 | 3.0 | 0 | 0.75 | 0 | 5.1e-15 |
| *X_3_* | 3.0 | 0 | 0.75 | 0 | 5.0 | 0 | 0 | 0.5 | 3.4e-16 |

Table 45 – Result of the 3-dimensional linear topology system case study.

## Gradient equations of the cost function

Let *X_j_*(*t_n_*) *j*=1,2,…,*M* and *n*=1,2,…,*N* be a point of the state variable *X_j_* at time *t_n_* and *S_j_*(*t_n_*) its respective slope value. According with the S-system theory described in the main manuscript, we can define the following vectors and matrices:

Production term vector

$PT_{i}(t_{n})=\alpha_{i}\prod_{j=1}^{M} X_{j}(t_{n})^{g_{ij}}$ (7)

Degradation term vector

$DT_{i}(t_{n})=\beta_{i}\prod_{j=1}^{M} X_{j}(t_{n})^{h_{ij}}$ (8)

Production parameter vector

$Vp_{i}=[\log\alpha_{i} g_{i1} g_{i2} \cdots g_{iM}]$ (9)

Degradation parameter vector

$Vd_{i}=[\log\beta_{i} h_{i1} h_{i2} \cdots h_{iM}]$ (10)

Regression matrix

$L=\left[\begin{matrix} 1 & \log(X_{1}(t_{1})) & \cdots& \log(X_{i}(t_{1})) & \cdots& \log(X_{M}(t_{1})) \\ 1 & \log(X_{1}(t_{2})) & \cdots& \log(X_{i}(t_{2})) & \cdots& \log(X_{M}(t_{2})) \\ \vdots& \vdots& \cdots& \vdots& \cdots& \vdots\\ 1 & \log(X_{1}(t_{N})) & \cdots& \log(X_{i}(t_{N})) & \cdots& \log(X_{M}(t_{N})) \end{matrix}\right]$ (11)

Resulting from the numerical decoupling, the following algebraic equation can be written

$S_{i}(t_{n})=PT_{i}(t_{n})-DT_{i}(t_{n}), n=1,2,\cdots,N$ (12)

$\log\left(PT_{i}\right)=\log\left(S_{i}+DT_{i}\right)$ (13)

After linearization of the algebraic system resulting of the numerical decoupling, the following vector can be defined for all time points:

$L\cdot Vp_{i}=y_{i}$, (14)

where

$y_{i}=\log\left(S_{i}+DT_{i}\right)$. (15)

Thus, the production parameter vector can be written as

$Vp_{i}=\left(L^{T}L\right)^{-1}L^{T}y_{i}$ (16)

Replacing 16 in 14 yields

$L\left(L^{T}L\right)^{-1}L^{T}y_{i}=y_{i}$. (17)

Defining the matrix *W*

$W=L\left(L^{T}L\right)^{-1}L^{T}$, (18)

and the vector

$\hat{y}_{i}=Wy_{i}$, (19)

the following cost function can be written

(20)

or

$\begin{matrix} F=\log\left(\left(Wy_{i}-y_{i}\right)^{T}\left(Wy_{i}-y_{i}\right)\right) \\ \\ =\log\left(\left((W-I)y_{i}\right)^{T}\left((W-I)y_{i}\right)\right) \end{matrix}$ (21)

The gradient of the cost function (Equations 20 and 21) with respect to the system parameters can be obtained as

$\frac{\partial F}{\partial\beta_{i}}=\frac{1}{\phi}\frac{\partial\left(\left((W-I)y_{i}\right)^{T}\left((W-I)y_{i}\right)\right)}{\partial\beta_{i}}$. (22)

Applying the rule chain yields

$\frac{\partial\left(\left((W-I)y_{i}\right)^{T}\left((W-I)y_{i}\right)\right)}{\partial\beta_{i}}=2\left(\frac{\partial\left((W-I)y_{i}\right)}{\partial\beta_{i}}\right)^{T}\left((W-I)y_{i}\right)$ (23)

$\frac{\partial\left((W-I)y_{i}\right)}{\partial\beta_{i}}=(W-I)\frac{\partial y_{i}}{\partial\beta_{i}}$ (24)

$\begin{matrix} \frac{\partial y_{i}}{\partial\beta_{i}}=\frac{\partial\log\left(S_{i}+DT_{i}\right)}{\partial\beta_{i}} \\ =\frac{1}{\left(S_{i}+DT_{i}\right)}\frac{\partial\beta_{i}\prod_{j=1}^{M} X_{j}(t_{n})^{h_{ij}}}{\partial\beta_{i}} \\ \\ =\frac{\prod_{j=1}^{M} X_{j}(t_{n})^{h_{ij}}}{\left(S_{i}+DT_{i}\right)} \\ \\ =\left(\prod_{j=1}^{M} X_{j}^{h_{ij}}\right)\circ\left(S_{i}+DT_{i}\right)^{\circ-1} \end{matrix}$ (25)

Finally, the gradient of *F* with respect to *β_i_* is

$\frac{\partial F}{\partial\beta_{i}}=\frac{2}{\phi}\left[\left(W-I\right)\left(\left(\prod_{j=1}^{M} X_{j}^{h_{ij}}\right)\circ\left(S_{i}+DT_{i}\right)^{\circ-1}\right)\right]^{T}\left[\left(W-I\right)\log\left(S_{i}+DT_{i}\right)\right]$. (26)

Similarly, the gradient with respect to the kinetic orders *h_ij_* can be obtained as

$\begin{matrix} \frac{\partial y_{i}}{\partial h_{ij}}=\frac{\partial\log\left(S_{i}+DT_{i}\right)}{\partial h_{ij}} \\ =\frac{1}{\left(S_{i}+DT_{i}\right)}\frac{\partial\beta_{i}\prod_{j=1}^{M} X_{j}(t_{n})^{h_{ij}}}{\partial h_{ij}} \\ \\ =\frac{\beta_{i}\prod_{j=1}^{M} X_{j}^{h_{ij}}\circ\log\left(X_{j}\right)}{\left(S_{i}+DT_{i}\right)} \\ \\ =\left(\beta_{i}\prod_{j=1}^{M} X_{j}^{h_{ij}}\circ\log\left(X_{j}\right)\right)\circ\left(S_{i}+DT_{i}\right)^{\circ-1} \end{matrix}$ (27)

which results in

$\frac{\partial F}{\partial h_{ij}}=\frac{2}{\phi}\left[\left(W-I\right)\left(\left(\beta_{i}\prod_{j=1}^{M} X_{j}^{h_{ij}}\circ\log\left(X_{j}\right)\right)\circ\left(S_{i}+DT_{i}\right)^{\circ-1}\right)\right]^{T}\left[\left(W-I\right)\log\left(S_{i}+DT_{i}\right)\right]$ (28).

The symbol ◦ represents the Hadamard product and $\left[v\right]^{\circ-1}=\left[\frac{1}{v_{i}}\right]$ is the Hadamard inverse operation for a given vector *v*.

## Software application

The implementation of the algorithm described in this report is provided both with open source (MathWorks Matlab) and as a stand alone application at <http://code.google.com/p/s-system-inference/> with free access and use, under a GNU GPL license.

Installation – computers without Matlab

1 – Go to http://bioinformaticstation.org/

2 – Download and run the MCRInstaller.exe

3 – Go to <http://code.google.com/p/s-system-inference/>

4 – Download Explore_S_system.exe and Explore_S_system.ctf

5 – Double click Explore_S_system.exe to start it.

Note: MCR only works if you are connected to the internet.

The Matlab script files are also provided.

1 – Download Explore_S_system.zip from <http://code.google.com/p/s-system-inference/>

2 – Use main_function.m within Matlab

Function description

Result=main_function(TS,S,Beta,Met,lbH,ubH,lbB,ubB,iter)

Intput:

TS_tp x m_ -time series of the state variable (tp – time points ; m –number of metabolites or state variables )

S_tp x 1_- Slope vector of one state variable

Beta – initial guess for beta

Met_1 x m_ – dependent state variable (*e.g.*, Met=[1 2 3 4] )

lbH – low boundary value of the kinetic parameters h

ubH – up boundary value of the kinetic parameters h

lbB – low boundary value of the constant rate Beta

ubB – up boundary value of the constant rate Beta

Output

Result.Alfa

Result.g

Result.Beta

Result.h

Result.error

## References

1. Kutalik Z, Tucker W, Moulton V: **S-system parameter estimation for noisy metabolic profiles using newton-flow analysis**. *IET Syst Biol* 2007, **1**(3):174-180.

2. Voit EO, Almeida J: **Decoupling dynamical systems for pathway identification from metabolic profiles**. *Bioinformatics* 2004, **20**(11):1670-1681.

3. Kikuchi S, Tominaga D, Arita M, Takahashi K, Tomita M: **Dynamic modeling of genetic networks using genetic algorithm and S-system**. *Bioinformatics* 2003, **19**(5):643-650.

4. Kimura S, Ide K, Kashihara A, Kano M, Hatakeyama M, Masui R, Nakagawa N, Yokoyama S, Kuramitsu S, Konagaya A: **Inference of S-system models of genetic networks using a cooperative coevolutionary algorithm**. *Bioinformatics* 2005, **21**(7):1154-1163.

5. Voit EO: **Computational analysis of biochemical systems : a practical guide for biochemists and molecular biologists**. Cambridge ; New York: Cambridge University Press; 2000.
